# Effect of *Crocus sativus* L. (saffron) and *Rosmarinus officinalis* L. (rosemary) in hepatocellular carcinoma: A narrative review of current evidence and prospects

**DOI:** 10.22038/ijbms.2025.84172.18204

**Published:** 2025

**Authors:** Gol Afrouz Naraki, Homa Fazeli Kakhki, Farid Qoorchi Moheb Seraj, Karim Naraki, Mahboobeh Ghasemzadeh Rahbardar

**Affiliations:** 1 Department of Orthotics and Prosthetics, School of Rehabilitation Science, Shiraz University of Medical Sciences, Shiraz, Iran; 2 Department of Pharmacodynamics and Toxicology, School of Pharmacy, Mashhad University of Medical Sciences, Mashhad, Iran; 3 Endovascular Section, Neurosurgical Department, Ghaem Hospital, Mashhad University of Medical Sciences, Mashhad, Iran; 4 Clinical Research Development Unit, Shahid Hasheminejad Hospital, Mashhad University of Medical Sciences, Mashhad, Iran

**Keywords:** Apoptosis, Carcinogenesis, Carnosol, Cell proliferation, Crocin, Liver neoplasms, Phytotherapy, Rosmarinic acid, Safranal

## Abstract

Hepatocellular carcinoma (HCC) is a considerable worldwide health concern that requires novel therapeutic approaches. Herbal therapy, with its rich historical origins and diverse pharmacological properties, provoked interest in its possible involvement in HCC treatment. *Crocus sativus* L. (saffron) and *Rosmarinus officinalis* L. (rosemary) are two frequently employed herbs in traditional Persian medicine for hepatoprotective properties. As a result, this review article aims to investigate the present landscape of therapies using saffron and rosemary, as well as their main components in the management of HCC. A thorough search was undertaken on Google Scholar, PubMed, and Web of Science for* in vivo *and *in vitro* studies on the effects of these two herbs on HCC. No time limitations were imposed, with the search extending until December 2024. Saffron and rosemary have shown promising anticancer activities against HCC through several mechanisms for instance, increasing apoptosis, adenosine monophosphate-activated protein kinase (AMPK) activation, decreasing colony formation, nuclear factor kappa B (NF-κB) and signal transducer and activator of transcription 3 (STAT3) activation, reducing the activity of Janus kinases (JAK)1, JAK2, protein kinase B (Akt)/mammalian target of rapamycin (mTOR) signaling pathway, as well as lowering vascular endothelial growth factor (VEGF) amounts in preclinical fields. While research into saffron, rosemary, and their main components in controlling HCC shows promise, it is critical to note that the belief that herbal therapy is always safe can be misleading. Caution is recommended while evaluating these approaches, as their effects and interactions with standard treatments may differ. More thorough clinical studies are needed to evaluate the safety and efficacy profiles of these herbal medicines in HCC thoroughly.

## Introduction

Liver cancer is still a primary global health concern, and its prevalence is rising globally (1, 2). It is predicted that by 2025, more than one million people will be diagnosed with liver cancer each year (3). Approximately 90% of liver cancer cases are attributed to hepatocellular carcinoma (HCC), the most prevalent kind of the disease. At around 50% of cases, hepatitis B virus (HBV) infection is the most common risk factor for the development of HCC (4). Since patients receive sustained virological response with antiviral medications, the risk associated with hepatitis C virus (HCV) infection has significantly decreased (5). Even after HCV suppression, people with cirrhosis continue to be at a significant risk of developing HCC. Particularly in the West, nonalcoholic steatohepatitis (NASH), which is linked to metabolic syndrome or diabetic mellitus, has been identified as the HCC etiology with the highest rate of growth (6). In addition, tobacco and aristolochic acid have been identified as possible pathogenetic cofactors in HCC by indicating mutational signatures (7). Aflatoxin intake, obesity, type 2 diabetes, nonalcoholic fatty liver disease (NAFLD), heavy alcohol use, and aflatoxins are further established risk factors for HCC (8).

Effective treatment and early diagnosis of HCC pose significant challenges. Most patients are asymptomatic until the disease advances. However, symptoms such as anorexia, bone pain, early satiety, fever, lethargy, obstructive jaundice, abdominal pain, watery diarrhea, and weight loss may manifest in some patients (8-10). 

Early detection is the key to treating HCC, which has an advantageous long-term prognosis (10, 11). The main treatment options for HCC are surgical resection or liver transplantation if the patient is a good candidate for a liver transplant. Nonetheless, a surgical cure is no longer an option for the great majority of HCC patients whose illness is discovered at an advanced stage (10). Chemotherapy is consequently required for the majority of patients. The most widely used chemotherapeutic treatment to treat HCC is sorafenib. This tiny multi-tyrosine kinase inhibitor inhibits the activity of Raf kinase, vascular endothelial growth factor (VEGF), and platelet-derived growth factor (PDGF) receptors (12). Despite being a targeted chemotherapy medicine, its use has only been demonstrated to slightly improve patient survival (13) by nearly 7–10 months (14). Targeting the broad angiogenic network observed in the liver, other medications, including sunitinib, brivanib, and other angiogenic inhibitors, are presently being developed and show promise (8). Levatinib is another first-line medicine for treating HCC, whereas regorafenib is an additional multi-kinase inhibitor recently approved for treating HCC. Regorafenib was developed as a subsequent treatment following sorafenib. Nevertheless, neither offers a significant advantage over sorafenib therapy (15, 16). Better therapeutic options are, therefore, still required.

Herbal treatments have attracted the interest of researchers and scientists across various fields, including those studying nervous system disorders (17, 18), malignancies (19, 20), and liver diseases (21), for centuries. Many herbal treatments or their primary constituents, including *Nigella sativa* (22), *Silybum marianum* (23), *Ginkgo biloba* (24), *Curcuma longa* (25), alpha-mangostin (26), and zeaxanthin (27) have hepatoprotective characteristics, which protect the liver from damage, promote regeneration (28), and exert anticancer effects (29) through mechanisms such as apoptosis induction, cell cycle arrest (30), and antiangiogenic activity (31). Nonetheless, it is critical to remember that the natural origin of these substances does not necessarily indicate superiority or the absence of possible adverse effects. Exploring the pharmacological mechanisms that drive these herbal medicines might provide valuable insights into their efficacy and safety as medical products for HCC management.

This study aims to advance therapy options for HCC through the investigation into the roles of *Crocus sativus* L. (saffron) and *Rosmarinus officinalis* L. (rosemary), two well-known herbs in Persian traditional medicine for their hepatoprotective properties and long history in folk medicine practices. The narrative review attempts to investigate the modern physio-pharmacological effects of these plants, offering light on their therapeutic potential as complementary or single treatments. The study aims to understand saffron and rosemary’s fundamental mechanisms of action and their main components in managing and controlling HCC by reviewing available literature. It also highlights the critical need for safer and more effective treatment alternatives for this challenging malignancy.

It is important to note that the following sections will explain in greater detail the significance of saffron and rosemary in traditional medicine and their current physio-pharmacological effects.

## Methods

Our objective in conducting an extensive investigation was to offer a detailed overview and assessment of the current knowledge base on the topic. Our team conducted a thorough search utilizing relevant search phrases linked to HCC, saffron, and rosemary on Google Scholar, PubMed, and Scopus. The keywords used were “*Crocus sativus* L.,” “saffron,” “crocin,” “crocetin,” “safranal,” “*Rosmarinus officinalis* L.,” “rosemary,” “rosmarinic acid,” “carnosic acid,” “carnosol,” “1,8-cineole,” “hepatocellular carcinoma,” and “HCC”. The search was carefully carried out to ensure that all relevant research was included, with no time limits imposed until December 2024. This analysis encompassed scholarly articles that explored the effectiveness of saffron or rosemary in managing HCC. The selection criteria comprised studies focusing on the mechanisms and effectiveness of these herbs and their main components on HCC, specifically emphasizing works accessible in English (at least in abstract form). Studies lacking peer review and works not pertinent to the aim of our study were excluded.

### The underlying mechanisms of HCC

The malfunction of a multi-step biological process in the liver that causes healthy hepatocytes to turn malignant leads to HCC development. HCC is caused by several variables, including immunology, inflammation, genetic and epigenetic changes, and control of oxidative stress, among others. Major risk factors for HCC include aflatoxins, hepatitis B and C, steatohepatitis triggered by drugs and alcohol, and carcinogens such as chlordane, pyrethrins, dithiothreitol, polychlorinated biphenyls, and polyvinyl chloride. These risk factors frequently impact hepatocytes, resulting in hepatotoxicity through cirrhosis, fibrosis, fatty liver, and cholestasis, which may lead to HCC (32). The development of HCC involves a complex interplay of biological mechanisms. Factors such as immunology, inflammation, genetic and epigenetic changes, and control of oxidative stress influence the progression from preneoplastic lesions to hepatic neoplasms (28, 33, 34). Inflamed or injured liver tissues, often due to persistent viral infections or exposure to toxins, create an inflammatory environment that fosters genetic alterations in hepatocytes, promoting their survival and proliferation (35, 36). 

Various risk factors influence oxidative stress, cell proliferation, cell death, mitochondrial function, lipid processing, insulin sensitivity, and more by controlling different cellular signaling pathways like P53, P73, Ras, wingless-related integration site (Wnt)/β-catenin, Janus kinase/signal transducer and activator of transcription 3 (JAK/STAT3), B-cell lymphoma 2 (Bcl-2), cyclin-dependent kinase 4 (CDK-4), mitogen-activated protein kinase (MAPK), tumor necrosis factor (TNF)-α, and transforming growth factor (TGF)-β, contributing to HCC progression (32).

Research into the molecular basis of HCC reveals the role of genetic, epigenetic, and signaling system abnormalities, emphasizing the need to understand these mechanisms in developing successful HCC treatments. 

**Figure 1:**
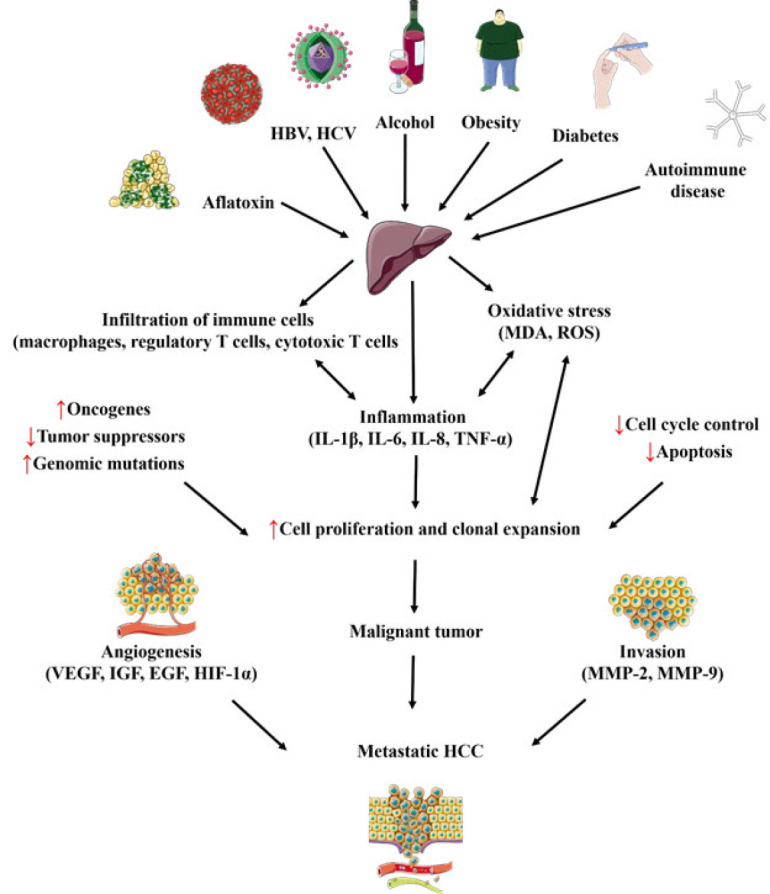
Molecular and cellular underlying mechanisms of HCC (images from https://smart.servier.com)

Hepatocarcinogenesis progresses from preneoplastic lesions to hepatic neoplasms by a complex interaction of biological mechanisms such as tumor microenvironment alterations, necroinflammation, oxidative stress, and hypoxia. This process also involves molecular mechanisms such as cytokine, chemokine, growth factor activation, DNA damage, and DNA methylation. HCC is primarily associated with inflammation, with more than 90% of cases occurring in inflamed or injured liver tissues as a result of persistent viral infections (HBV, HCV), aflatoxin exposure, or alcohol intake (Figure 1). Cytokines, chemokines, and growth factors provide an inflammatory environment that promotes hepatocyte genetic alterations. Transformed hepatocytes survive and proliferate by activating anti-apoptotic pathways and escaping immune detection. The complex interaction of pro-inflammatory (e.g., interleukin (IL)-6, TNF-α) and anti-inflammatory cytokines (e.g., TGF-α and TGF-β), as well as proteins like nuclear factor kappa B (NF-κB), STAT-3, and their signaling pathways, contribute to the development of HCC (37-39).

IL-6 and TNF-α activate STAT3 during chronic hepatic injury, promoting neoplastic transformation (38). TNF-α also fuels hepatic tumor growth and HCC recurrence (39). NF-κB drives inflammation and cell death, which is crucial in HCC development (40, 41). TGF-β, upregulated post-injury, fosters HCC progression by promoting cell proliferation, dysplasia, and invasion (42).

HCC development also includes accumulating genetic and epigenetic changes throughout initiation, promotion, and progression. Telomere shortening, copy number variants, single nucleotide variants, and epigenetic changes are all significant molecular aberrations in HCC. Telomere shortening causes changes in gene expression via mutations and chromosomal abnormalities. In contrast, copy number variants involve mutations in tumor suppressor genes like p53 and proto-oncogenes like Ras and c-myc. Epigenetic alterations, such as inappropriate DNA methylation patterns, inhibit gene expression, affecting tumor suppressor genes such as p16INK4A and E-cadherin (39).

Furthermore, research on tumor signal transduction pathways indicates the importance of multiple pathways in HCC progression. The Wnt/β-catenin pathway is essential for maintaining cellular homeostasis and regulating activities like proliferation and differentiation. Mutations in β-catenin can cause constitutive activation of β-catenin/T cell factor (TCF) complexes, increasing HCC development (43-45). Activation of this system is linked to HCV infection and aflatoxin B1 exposure (46, 47). 

Another important pathway is the rat sarcoma (Ras)/rapidly accelerated fibrosarcoma (Raf)/ MAPK pathway, essential for cell proliferation, growth, and survival. Dysregulation caused by aberrant signals and viral infections can lead to cancer (45). 

In addition, the phosphatidylinositol 3-kinase (PI3)/protein kinase B (Akt)/mammalian target of rapamycin (mTOR) pathway regulates cell proliferation, metabolism, and survival. In a large proportion of HCC patients, activation of this pathway inhibits apoptosis and promotes tumor development (45, 48). Besides, the JAK/STAT system, which is involved in differentiation, proliferation, and apoptosis, has abnormal activity in HCC, with active STAT3 associated with aggressive tumors (43).

Moreover, the ubiquitin-proteasome (UP) pathway degrades cellular proteins and is critical to maintaining cellular homeostasis. Overexpression of Gankyrin in HCC can cause accelerated cell proliferation and the degradation of tumor suppressor proteins (39, 49, 50). Angiogenesis is also important in HCC, as the tumor relies largely on vessel formation to progress. High levels of VEGF, angiopoietins, basic fibroblast growth factor (bFGF), TGF-β, and insulin-like growth factor II (IGF-II) promote vascular expansion and have been associated with a poorer prognosis in HCC (46-48). Understanding these pathways and variables is important in developing successful treatments for HCC.

### C. sativus L. effects on HCC

**Figure 2:**
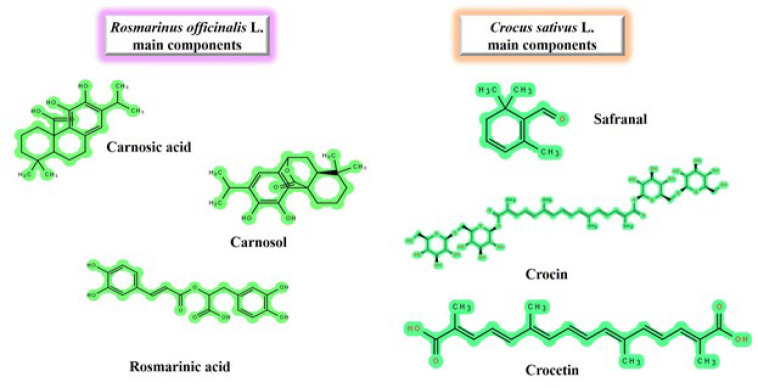
Main components of *Crocus sativus* and *Rosmarinus officinalis*

*C. sativus* L., scientifically known as saffron, is a perennial plant primarily grown in Iran and a member of the Iridaceae family. Saffron has traditionally been used in herbal medicine for various purposes, including as an expectorant, aphrodisiac, eupeptic, antispasmodic, diaphoretic, carminative, and anticatarrhal (51). This herb is a potent hepatic deobstruent and liver tonic. According to Tabari, saffron has hepatoprotective properties that are “warm, moderate, and dry.” It is bitter and resolvent. As a result, it can cure liver obstructions (52). Furthermore, Jingzhu Materia Medica, a traditional Tibetan medicine, documented the use of saffron in liver illnesses, stating that “saffron can be used to treat all liver diseases” (53). In addition to its use as a food colorant and spice, saffron is also known for its varied pharmacological effects, which are primarily attributed to three important constituents: crocin (crocetin glycoside), safranal, and crocetin (51) (Figure 2). Furthermore, pharmacological investigations disclosed other effects of this herb including anti-oxidant (54, 55), anti-apoptotic (56), anti-inflammatory (57), anti-obesity (58), cytoprotective (59), antiasthmatic (60, 61), renoprotective (62, 63), and neuroprotective (64) properties. 

### In vitro

*C. sativus extracts*

*C. sativus* aqueous extract had a cytotoxic effect on HepG-2 cells by decreasing nitric oxide (NO) concentration (65). *C. sativus* aqueous-ethanolic extract was shown to reduce NF-κB activation, enhance caspase-3 cleavage, cause DNA damage, and induce cell cycle arrest in HepG2 cells (66). An *in vitro* study aimed to find out how saffron affected the liver cancer cell line QGY-7703. The findings showed that saffron administration successfully inhibited QGY-7703 cell growth, stopped the cell cycle at the G0/G1 phase, and caused apoptosis. Furthermore, telomerase activity and human telomerase reverse transcriptase (hTERT) levels in these cells were decreased by saffron treatment. Additionally, after saffron therapy, there were noticeable alterations in cell shape, a rise in senescent cells, an enhanced Bcl-2-associated protein x (Bax)/Bcl-2 ratio, and increased P21 expression (67). 

*Crocetin*

A study was designed to find out how crocetin affected HCC cells. It was discovered that crocetin decreased the invasive potential of HCC cells, inhibited cell proliferation, and induced apoptosis. Notably, without influencing STAT5 activation, crocetin inhibited constitutive/inducible STAT3 activation, prevented STAT3 nuclear accumulation, and decreased its DNA binding activity in HCC cells. Additionally, the treatment reduced the activity of upstream kinases such as JAK1, JAK2, and Src. Tyrosine phosphatases were implicated in reversing the crocetin-induced STAT3 suppression upon treatment with sodium pervanadate. Src homology region 2 domain-containing phosphatase-1 (SHP-1), a crucial role in regulating cellular signaling pathways, expression was elevated by crocetin, and the suppression of STAT3 by crocetin was reversed by small interfering ribonucleic acid (siRNA) silencing SHP-1. Additionally, STAT3-regulated genes such as Bcl-2, B-cell lymphoma-extra-large (Bcl-xL), cyclin D1, survivin, VEGF, cyclooxygenase-2 (COX-2), and matrix metalloproteinase (MMP)-9 were suppressed after crocetin administration (68). 

In another research, magnetic nanoparticles coated with poly (ethylene glycol) were produced successfully and then loaded with different doses of crocetin. *In vitro*, these crocetin-coated pegylated magnetic nanoparticles showed strong anti-proliferative effects on HepG2 cells with favorable release kinetics at various pH levels. With estimated half-maximal inhibitory concentration (IC_50_) values of 0.1019, 0.0903, and 0.0462 mg/ml for varying doses of crocetin- poly (ethylene glycol)-magnetic nanoparticles, the improved drug delivery system demonstrated notable inhibitory effects on HepG2 cell proliferation. (69). 

*Crocin*

Crocin revealed cytotoxic effects on HepG2 cells by decreasing telomerase activity and reducing the expression level of the catalytic subunit of the hTERT gene (70). *In vitro* studies on HepG2 cells indicated that crocin arrested the cell cycle at specific phases, induced apoptosis, and decreased inflammation (71). A study aimed to use magnetite nanoparticles coated with crocin to provide a novel therapy strategy for liver cancer. When compared to uncoated magnetite nanoparticles, free crocin, or controls, treatment with crocin-coated magnetite nanoparticles dramatically reduced the proliferation of HepG2 cells (72).

The objective of a study was to clarify how autophagy contributes to crocin-induced apoptosis in HCC. Crocin inhibited growth by inducing apoptosis in a dose- and time-dependent manner. As soon as six hours after treatment, HepG2 and HCCLM3 cells showed elevated microtubule-associated protein 1A/1B-light chain 3 (LC3) puncta and upregulated LC3-II accumulation, which were early indicators of autophagy. Furthermore, autophagy preceded apoptosis in HCC cells exposed to crocin, indicating a sequential link between autophagy onset and apoptosis activation. Autophagy suppression by 3-methyladenine prevented these cells from undergoing crocin-induced apoptosis. As demonstrated by decreased activity of important proteins such as p-Akt (S473), p-mTOR (S2448), and p-p70S6K (T389), crocin inhibited the Akt/mTOR signaling pathway, suggesting an Akt/mTOR-dependent mechanism for crocin-induced autophagic death in HCC cells. The complex interaction between autophagy and apoptosis in crocin treatment in HCC was further highlighted by the observation that forced expression of Akt, which inhibited autophagy, also inhibited crocin-induced apoptosis in HCC cells (73). The effect of crocin on the IL-6/STAT3 signaling pathway was investigated to see whether it could inhibit the growth of liver cancer cells and cause them to undergo apoptosis. By specifically targeting JAK1, JAK2, and Src kinase, crocin efficiently decreased IL-6-induced STAT3 activation in Hep3B and HepG2 cells, thereby preventing STAT3 phosphorylation. Crocin also increased the production of SHP-1, which dephosphorylated STAT3. The inhibitory effects of crocin were reversed when the SHP-1 gene was silenced by siRNA, highlighting the crucial function that SHP-1 plays in this process. Furthermore, in correlation with increased apoptosis and decreased proliferation, crocin increased levels of the pro-apoptotic protein Bax while downregulating the expression of angiogenic (VEGF), invasive (C-X-C chemokine receptor type 4 (CXCR-4)), proliferative (cyclin D1), and STAT3-regulated anti-apoptotic (Bcl-2, survivin) proteins (74).

On HepG2 cells, crocin plus sorafenib demonstrated a synergistic antitumor activity (75). A study assessed the synergistic effects of crocin nanoparticles on human HepG2 and non-cancerous cells (WI38) when combined with doxorubicin treatment. Compared to native crocin, crocin nanoparticles had considerably stronger antitumor activity against HepG2 cells, with lower IC_50_ values. HepG2 cells treated with crocin nanoparticles exhibited the highest apoptosis and cell cycle arrest rates at the G2/M phase. Apoptotic and autophagic genes were shown to be upregulated in doxorubicin/crocin nanoparticles co-treatment, according to gene expression analysis. On the other hand, WI38 cells did not react negatively to crocin nanoparticles but were more sensitive to doxorubicin toxicity (76). 

*Safranal*

Researchers investigated the anticancer effects of safranal against HCC. The findings showed that safranal significantly affected DNA damage repair processes while inducing cell cycle arrest at various phases, notably in the G2/M phase at 6 and 12 hr and at the S-phase at 24 hr. Furthermore, by triggering either extrinsic or intrinsic initiator caspases, safranal showed pro-apoptotic effects, indicating that endoplasmic reticulum stress pathways may be involved in the induction of apoptosis. Following safranal treatment, gene set enrichment analysis revealed the elevation of genes linked to the unfolded protein response. This suggests that endoplasmic reticulum stress modulation plays a role in safranal-induced responses in HepG2 cells (77). 

**Figure 3:**
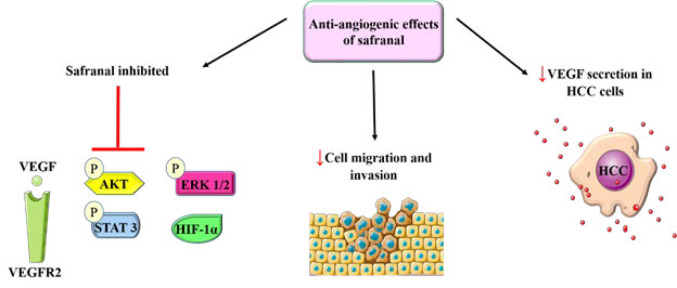
Antiangiogenic effect of safranal on HCC (images from https://smart.servier.com)

An investigation of the antiangiogenic effects of safranal on HCC was conducted. Because the vascular supporting system is less adaptive than malignant cells, targeting it instead of the tumor cells themselves is an appealing strategy. The study effectively showed that VEGF secretion of HepG2 cells was suppressed. Additionally, safranal lowered the expression of signaling molecules such as p-AKT, p-extracellular signal-regulated kinase (ERK)1/2, MMP9, p-focal adhesion kinase (FAK), and p-STAT3, as well as important angiogenesis-related proteins like hypoxia-inducible factor 1alpha (HIF-1α), VEGF, and VEGF receptor (VEGFR)2 (78) (Figure 3). The chemopreventive properties of safranal on the human liver cancer cell line HepG2 were examined in a study. It was observed that safranal reduced inflammation and caused apoptosis in HepG2 cells (79).

A study reported that safranal caused extensive DNA damage and protein destabilization by inducing oxidative stress in HepG2 cells (80).

### In vivo

*C. sativus extracts*

A study investigated the efficacy of *C. sativus* aqueous-ethanolic extract in preventing diethylnitrosamine-induced liver cancer in rats. *C. sativus* aqueous-ethanolic extract significantly decreased placental glutathione S-transferase-positive foci and diethylnitrosamine-induced hepatic dyschromatic nodules in rat livers. Additionally, by lowering myeloperoxidase activity, malondialdehyde (MDA), and protein carbonyl production in the liver and raising superoxide dismutase (SOD), catalase (CAT), and glutathione-S-transferase levels, *C. sativus* aqueous-ethanolic extract mitigated diethylnitrosamine-induced oxidative stress. The extract inhibited NF-κB p-65, COX-2, inducible nitric oxide synthase (iNOS), Ki-67, and phosphorylated TNF receptors. In the liver tissues of rats given diethylnitrosamine, the extract also stopped the decline of terminal deoxynucleotidyl transferase dUTP nick end labeling (TUNEL) and M30 CytoDeath-positive cells (66).

In an experimental HCC rat model, the tumor-suppressive properties of *C. sativus* aqueous extract were investigated. Three groups of rats were formed: The control group, which was given only distilled water; the HCC group, which was given diethylnitrosamine and CCl_4_; and the *C. sativus* aqueous extract group, which was given *C. sativus* aqueous extract orally for two weeks before the beginning of HCC and for six weeks after that. According to the results, *C. sativus* aqueous extract administration significantly reduced hepatic dyschromatic nodules, increased albumin levels, decreased TGF-β levels, increased total anti-oxidant capacity levels, and lowered alanine transaminase (ALT) and aspartate aminotransferase (AST) activities. Additionally, *C. sativus* aqueous extract treatment improved liver and kidney health by restoring hematological indicators to those of the negative control group (81). The administration of *C. sativus* aqueous extract to rats with diethylnitrosamine-induced HCC. The obtained data disclosed that *C. sativus* aqueous extract restored the levels of CAT, glutathione (GSH), and SOD. Moreover, it elevated active caspase-3 expression, prevented MMP-9 production, and decreased the diethylnitrosamine-induced rise in the incidence and number of hepatic dyschromatic nodules (82).

*Crocin*

The effects of green synthesized silver nanoparticles from *C. sativus* on rats’ liver tissues that had pre-HCC triggered by diethylnitrosamine were studied. Five groups of rats were formed. 1 (control), 2 (Diethylnitrosamine as a single dosage), 3 (silver nanoparticles made from *C. sativus*), 4 (Diethylnitrosamine plus silver nanoparticles made from *C. sativus*), and 5 (silver nanoparticles made from *C. sativus* plus diethylnitrosamine). According to the histopathological investigations, the hepatocytes in group 1 were normal. Group 2 displayed abnormal cells, fatty changes, severe necrosis, and bile duct proliferation. Hepatocytes of group 3 were normal, with only a little necrosis. Group 4 showed minor lipid changes, congestion, infiltration of inflammatory cells, and a specific area of necrosis; no unusual cells were visible. The liver segment of group 5 reveals a small area of necrosis, an infiltration of inflammatory cells, a slight fatty alteration, and no abnormal cells (83). A study aimed to investigate the potential chemopreventive effects of crocin against chemically-induced liver cancer in rats and to explore the mechanisms underlying its antitumor properties. The results revealed that crocin demonstrated anti-proliferative and pro-apoptotic effects in the induced-HCC model and anti-inflammatory properties by inhibiting NF-κB and other inflammatory markers (71).

The study aimed to develop a new treatment approach for liver cancer using magnetite nanoparticles coated with crocin. Histological analyses of the livers of mice given diethylnitrosamine injections showed several precancerous abnormalities, such as nuclear abnormalities, bile duct modifications, and hepatic foci. Increased cell proliferation and death indicators were seen by immunohistochemistry as precancerous lesions developed. Magnetite nanoparticles coated with crocin caused precancerous lesions to reduce, apoptotic cells to be upregulated, and indicators linked to angiogenesis, inflammation, oxidative stress, and cell proliferation to be downregulated (72). The chemopreventive potential of crocin against hepatocarcinogenesis in rats was investigated in a study that concentrated on its impact on the nuclear factor erythroid 2–related factor 2 (Nrf2) and apoptotic signaling pathways. Crocin treatment significantly reduced thioacetamide-induced malignant lesions while restoring compromised liver functions in rats given thioacetamide to induce hepatocarcinogenesis. This impact was associated with decreased Kelch-like ECH-associated protein 1 (Keap-1) and increased heme oxygenase-1 (HO-1) and Nrf2 expression in the liver. Crocin also strengthened anti-oxidant defenses and hepatic oxidative status. Additionally, crocin enhanced the expression of the p53 gene, upregulated the expression of caspase-8 and TNF-related apoptosis-inducing ligand (TRAIL), and decreased the levels of c-Jun N-terminal kinase (c-JNK). The effect of crocin on intrinsic apoptosis was highlighted by its notable influence on the balance of anti-apoptotic (Bcl-2) and pro-apoptotic (Bax) indicators (84).

The administration of crocin resulted in decreased weight loss, attenuated levels of C-reactive protein (CRP), IL-6, lactate dehydrogenase (LDH), oxidative stress markers, NF-κB, p53, TNF-α, and VEGF, as well as histopathological alterations in a rat model of HCC. Concurrent treatment with sorafenib and crocin improved inflammatory factors and histopathological parameters compared with independent therapies (75).

In a cirrhotic rat model of HCC, a study examined the therapeutic potential of crocin both by itself and in combination with sorafenib. After diethylnitrosamine exposure caused cirrhosis and HCC in rats, crocin and sorafenib, either separately or in combination, were administered after three weeks. Significantly, when compared to solo therapies, the combined therapy significantly decreased dyschromatic nodules, dysplastic nodules, and nodule multiplicity. Significant apoptosis was induced, proliferative cell and β-catenin levels in tumor tissues were reduced, and COX-2 and NF-κB levels were substantially decreased (85) (Table 1).

*Safranal*

The chemopreventive properties of safranal against diethylnitrosamine-induced liver cancer in rats were examined. In rats administered diethylnitrosamine, safranal significantly reduced proliferation (Ki-67) and induced death. It also showed anti-inflammatory properties by blocking important inflammatory markers, including COX-2, iNOS, NF-kB, TNF-α, and its receptor (79). 

In summary, the findings of the studies show that extracts from *C. sativus*, crocetin, crocin, and safranal have a substantial effect on liver cancer cells. *In vitro*, these compounds demonstrated cytotoxic effects, caused apoptosis, and altered important signaling pathways, indicating their potential as medications. Crocetin and crocin inhibited cell proliferation, induced apoptosis, and suppressed essential pathways such as STAT3 and Akt/mTOR in HCC cells. Specifically, crocetin was found to inhibit constitutive and inducible STAT3 activation, preventing its nuclear accumulation and DNA binding activity, which is crucial for the transcription of anti-apoptotic genes. This suppression of STAT3 is also correlated with decreased expression of downstream targets such as Bcl-2, cyclin D1, and survivin, which are known to promote cell survival and proliferation. Similarly, the modulation of the Akt/mTOR pathway by crocin suggests a mechanism by which it can induce autophagy and apoptosis, as evidenced by the upregulation of pro-apoptotic proteins like Bax and the downregulation of anti-apoptotic factors. Safranal also had anticancer properties, causing cell cycle arrest, apoptosis, and antiangiogenic effects in HCC cells. The mechanisms underlying the effects of safranal include the induction of endoplasmic reticulum stress, which activates both extrinsic and intrinsic apoptotic pathways, as well as the modulation of key angiogenic factors such as VEGF and HIF-1α, thereby inhibiting tumor growth and metastasis. Despite these promising findings, it is important to note that the methodological details, such as experimental conditions, were not consistently reported, raising concerns about the reproducibility and reliability of these results.

*In vivo* studies have discovered the efficiency of *C. sativus* extracts in preventing diethylnitrosamine-induced liver cancer in rats, demonstrating substantial benefits such as the reduction of hepatic dyschromatic nodules, oxidative stress mitigation, and regulation of key inflammatory markers. The extracts were shown to inhibit the NF-κB pathway, which is often activated in cancer and contributes to inflammation and cell survival. By reducing NF-κB activity, *C. sativus* extracts may help lower pro-inflammatory cytokine expression and promote apoptosis in cancerous cells. Crocin demonstrated substantial potential in treating chemically-induced liver cancer in rat models. It exerted anti-proliferative and pro-apoptotic effects, plus anti-inflammatory properties by targeting NF-κB and other inflammatory markers. Moreover, the ability of crocin to modulate the expression of the tumor suppressor gene p53 and apoptotic signaling pathways, including caspase-8 and TRAIL, further underscores its therapeutic potential. In the same way, safranal has been shown to have chemopreventive effects against diethylnitrosamine-induced liver cancer in rats by lowering proliferative markers and triggering cell death, as well as anti-inflammatory effects by targeting key inflammation markers. However, the variability in dosing regimens and treatment durations across studies necessitates a more standardized approach to enhance the reliability of these results. 

These results emphasize the potential of these compounds to address liver cancer through various mechanisms, thereby prompting further research into their therapeutic applications and potential synergies with existing treatments. However, these compounds’ long-term effects and safety profiles in human populations remain largely unexplored, highlighting a significant research gap. Future studies should aim to identify biomarkers to predict responses to saffron-based treatments, thereby personalizing therapy for HCC patients. Additionally, a deeper investigation into the molecular pathways involved in the action of these compounds will be crucial for optimizing their use in clinical settings and understanding their interactions with conventional therapies.

### Rosmarinus officinalis L. effects on HCC

*Rosmarinus officinalis* L. (*Salvia rosmarinus* Spenn), also known as rosemary, is a constantly green bushy plant that thrives in the sub-Himalayan regions and the Mediterranean sea. In traditional herbal remedies, practitioners have administered rosemary as a moderate analgesic and antispasmodic to treat depression, intercostal neuralgia, rheumatic pain, emotional upset, headaches, insomnia, dysmenorrhea, and migraines (86, 87). In traditional medicine, rosemary has also been extensively employed as a hepatoprotective and choleretic medication (88).

Alkaloids, diterpenes (such as rosmarinic acid and carnosic acid), flavonoids, monoterpene hydrocarbon-rich essential oils (such as alpha-pinene and camphor), phenolic acids, saponins, and tannins are some of the bioactive substances found in rosemary. These compounds possess diverse medicinal properties and contribute to their distinct fragrance (89).

Modern pharmacology revealed various properties for this herb and its main components like anti-inflammatory (90, 91), anti-oxidant (86), hypnotic (89), antirheumatic (92), antidote (93), antinociceptive (94), anti-obesity (58), cardioprotective (95, 96), antidepressants (97), and neuroprotective (98). Although rosemary is generally considered safe for food preservation, prolonged or excessive consumption may pose risks, potentially impacting the liver, kidneys, and reproductive health and causing developmental concerns (99). Furthermore, various research has demonstrated its hepatoprotective and anticancer properties, which will be described in the following section. 

### In vitro

*R. officinalis oil and extracts*

Treating HepG2 cells with *R. officinalis* oils enhanced Bax expression and lowered Bcl-2 expression (100). The effect of cytotoxic levels of *R. officinalis* oil on the cell cycle and its potential to trigger DNA fragmentation and apoptotic cell death in HepG2 cells was examined. Assessment of morphological alterations and micronucleus formation in HepG2 cells indicated no significant rise in micronuclei count in *R. officinalis* oil-treated cells compared to controls. *R. officinalis* oil prompted apoptosis-associated morphological changes in a concentration-dependent manner, with minimal necrosis observed. These changes included cytoplasmic membrane shrinkage and the development of apoptotic bodies. Moreover, higher concentrations of *R. officinalis* oil led to internucleosomal DNA fragmentation (formation of a DNA ladder) in these cells. Analysis of the cell cycle demonstrated cell accumulation in the G1 phase, accompanied by a decrease in the number of cells in the S phase following 24 hr of exposure to the substances under investigation. This decelerated or halted cell division, resulting in cell death (101).

Treating Hep-G2 cells with *R. officinalis* methanolic extract augmented glucose consumption, AMP-dependent kinase (AMPK) phosphorylation, and acetyl-CoA carboxylase (ACC). It also reduced the messenger ribonucleic acid (mRNA) level of glucose-6-phosphatase (G6Pase), PPARγ coactivator 1α (PGC1α), sirtuin 1 (SIRT1), and low-density lipoprotein receptor (LDLR), as well as expression of ACCB. GW9662, a PPARγ inhibitor, lessened the effects of *R. officinalis* extract on glucose consumption (102). To determine how rosemary extract might affect HepG2 cells, its anticancer activity was assessed. In HepG2 cells, rosemary extract increased the level of Nrf2 protein while also inducing the expression of sestrin2 and multidrug resistance-associated protein 2 (MRP2). It is reasonable to suppose that Nrf2 overexpression may cause the increased expression of Sestrin2 and MRP2, even if the activation mechanism of Nrf2/anti-oxidant response element (ARE) was not precisely evaluated (103).

*1,8-cineole*

A study assessed the mechanisms of action of 1,8-cineole as well as the possible advantages of combining it with anticancer medicines that exhibit “anti-senescence” effects in HepG2 cells.1,8-Cineole promoted G0/G1 arrest, which decreased cell proliferation. 1,8-cineole caused cellular senescence but was not able to cause apoptosis. ROS generation, ΔΨm depolarization, AMPK, ERK, p38 activation, and mTOR inhibition were all enhanced by 1,8-cineole. Vitamins and N-acetyl-L-cysteine, two anti-oxidants, inhibited 1,8-cineole-induced senescence and growth suppression in HepG2 cells. HepG2 cells became more susceptible to the anti-senescence drugs quercetin, simvastatin, SB202190, and U0126 through a pre-incubation with 1,8-cineole. Simvastatin and 1,8-cineole therapy together caused apoptosis, and combinations of the two compounds suppressed cell viability (104).

*Carnosic acid*

In an *in vitro* model of cellular fat accumulation, carnosic acid protected Hep-G2 cells by increasing the phosphorylation of MAPK and epithelium growth factor receptor (EGFR), besides lowering the levels of peroxisome proliferator-activated receptor gamma (PPARγ) level (105). Furthermore, pretreating Hep-G2 cells before exposure to H_2_O_2_ with carnosic acid enhanced cell viability, GSH, B-cell lymphoma-extra-large (Bcl-xL), manganese superoxide dismutase (MnSOD), SIRT1, while it attenuated LDH activity and caspase-3 (106).

On HepG2 cells, carnosic acid’s cytotoxic and apoptotic-inducing effects were examined. The findings showed that carnosic acid reduced HepG2 cell viability dose-dependently. Rapid caspase-3 activation and subsequent poly (ADP-ribose) polymerase (PARP) proteolytic cleavage were triggered by carnosic acid treatment, which was indicative of apoptotic cells. Moreover, the Bcl-2/Bax protein ratio was lowered, and mitochondrial membrane potential was dissipated by carnosic acid. In addition, carnosic acid decreased Akt phosphorylation (107). Similarly, another group of researchers indicated that treating SMMC-7721 and HepG2 cells with carnosic acid resulted in enhanced levels of apoptosis, augmented activities of caspase-3, caspase-8, and caspase-9, increased amounts of intracellular reactive oxygen species (ROS), decreased levels of cell viability, cell proliferation, migration, and reduced mitochondrial membrane potential (108). The potential impact of carnosic acid on HepG2 cells was investigated to assess its anticancer properties. It was observed that carnosic acid led to increased levels of Sestrin2 and MRP2 in HepG2 cells, along with a boost in Nrf2 protein expression. Notably, carnosic acid’s effect was more pronounced than rosemary extract and carnosol. While the specific activation pathway of Nrf2/ARE was not explicitly examined, it is likely that the upregulation of Sestrin2 and MRP2 could be attributed to the enhancement of Nrf2 expression (103).

In cancer cell lines, carnosic acid has been demonstrated to augment sorafenib-induced cell death, primarily due to a combination of promoted apoptosis and cytotoxic autophagy. Furthermore, adding carnosic acid enhanced the increase of ROS levels caused by sorafenib. It was shown that carnosic acid increased the lengthening of the cell cycle induced by sorafenib and that a general decline in cell growth was linked to a decrease in the activation of both ERK1/2 and STAT3 transcription factors (109). Furthermore, another research has demonstrated that supplementing carnosic acid with the vitamin D2 analog, doxercalciferol can increase the cytotoxic effect of sorafenib on the HCC cell lines HCO2, which is sorafenib-resistant, and Huh7, which is sorafenib-sensitive. This combination promoted both apoptosis and autophagy while elevating HCC cell death in cell lines. Increased cytoplasmic vacuolation, LC3) perinuclear aggregation, and higher protein levels of the autophagy markers Beclin1, autophagy-related 3 (Atg3), and LC3 all indicated autophagy (110).

Treating HepG2 cells exposed to forskolin with carnosic acid augmented the phosphorylation of acetyl-CoA carboxylase 1 (ACC1) and AMPK. They also reduced phosphoenolpyruvate carboxykinase 1 (PCK1), glucose-6-phosphatase (G6PC), mRNA levels of sterol regulatory element-binding protein 1c (SREBP-1c), fatty acid synthase (FAS), ACC1, cell proliferation, and cell viability (111). Additionally, treating MHCC97H cells with a carnosic acid nanocluster-based framework resulted in augmented levels of apoptosis and reduced amounts of migration, invasion, proliferation, and lowered activity of the Wnt/β-catenin signaling pathway. The authors also reported that the activity of CA-NBF was considerably greater than carnosic acid (112). 

*Carnosol*

Its possible effect on HepG2 cells was examined to evaluate the anticancer properties of carnosol. It was found that carnosol enhanced Nrf2 protein expression and raised Sestrin2 and MRP2 levels in HepG2 cells. Although an exact Nrf2/ARE activation route was not investigated, it is reasonable to assume that higher levels of Nrf2 are responsible for the overexpression of Sestrin2 and MRP2 (103). Using carnosol to treat HepG2 cells exposed to forskolin increased AMPK and ACC1 phosphorylation. Additionally, it decreased cell viability, PCK1, G6PC, FAS, ACC1, and SREBP-1c mRNA levels (111). Furthermore, carnosol regulated the AMPK signaling pathway to inhibit HepG2 and Huh7 cell proliferation, invasion, and migration (113).

*Rosmarinic acid*

Treating HepG2 cells with rosmarinic acid markedly reduced HepG2 cell viability. There were also indications of dose-dependent reductions in cell growth. Rosmarinic acid showed evidence of triggering apoptosis and promoting cell cycle arrest in G1. Sixteen differently expressed proteins were effectively identified by the proteomics study. The identified proteins were involved in multiple biological processes and displayed a variety of molecular roles, primarily associated with the inactivation of the glycolytic pathway. It also suppressed the production of lactate and adenosine triphosphate (ATP) as well as the uptake of glucose by downregulating the expression of hexokinase-2 and glucose transporter-1 (114). Combined treatment of HepG2 and Bel-7402 Cells with rosmarinic acid and doxorubicin caused a rise in damaged cell morphology, apoptosis, Bax expression, and DNA damage. In addition, the concurrent treatment of rosmarinic acid and doxorubicin attenuated cell viability, S-phase cell numbers, and Bcl-2 expression (115). 

Additionally, treating SMMC 7721 cells with rosmarinic acid enhanced G1 arrest and apoptosis, lessening cell proliferation and invasion (116). An investigation reported that treating HepG2 cells with rosmarinic acid increased anti-oxidant properties (hydroxyl radical scavenging activity), cell cycle arrest at the G0/G1 phase, cell death, the mRNA expression of Bax, ERK2, and decreased Bcl-2 mRNA expression (117). Researchers examined how rosmarinic acid affected apoptosis, autophagy, proteasome inhibition, cellular stressors, and MG132-induced cytotoxicity in HepG2 cells. In HepG2 cells, the highest dose of rosmarinic acid reduced cell viability. A substantial increase in the amounts of activating transcription factor 4 (ATF4), binding immunoglobulin protein (BiP), heat shock protein 70 (HSP70), LC3B-II, cleaved PARP1, polyubiquitinated protein, and protein carbonyl was observed in response to MG132. It also significantly reduced the cells’ viability and phosphorylated the rapamycin’s mammalian target. In MG132-treated cells, the highest dose of rosmarinic acid significantly reduced cell viability and increased levels of polyubiquitinated protein, LC3B-II, HSP70, BiP, ATF4, protein carbonyl, and cleaved PARP1 (118). Besides, rosmarinic acid augmented the apoptosis ratio by increasing the levels of Bax, caspase-3, and caspase-9 HepG2 cells and decreasing the amounts of Bcl-2. It also lessened cell viability, cell migration, and invasion in these cells (119). Also, exposing HepG2 cells to rosmarinic acid caused a significant enhancement in apoptosis rate, cleaved caspase-3, cytochrome C, Bax expression, and cell cycle arrest in the S phase. It also lowered cell viability, mobility, and Bcl-2 expression (120).

### In vivo

*R. officinalis extracts*

Treating rats with diethyl nitrosamine-induced hepatocellular carcinoma with *R. officinalis* ethanolic extract augmented serum total protein and albumin. It also lessened the amounts of ALT, AST, alkaline phosphatase (ALP), gamma-glutamyl transferase (GGT), LDH, and alpha-fetoprotein (AFP). *R. officinalis* ethanolic extract’s ameliorative effects were comparable to cisplatin (121).

*1,8 cineole*

The effects of 1,8 cineole have been assessed against diethylnitrosamine/2-acetylaminofluorene-induced HCC in rats. The results indicated that 1,8 cineole enhanced the functional capacity of the liver and hepatic miR-122 level. Besides, it decreased ferritin, alpha-L-fucosidase, arginase-1, alpha-fetoprotein, epithelial-mesenchymal transition (EMT), fascin-1 (FSCN1), MMP-9, TGF-β1, vascular endothelial growth factor (VEGF), and vimentin. The ameliorative effects of 1,8 cineole were comparable to doxorubicin (122).

*Carnosic acid*

The administration of carnosic acid nanocluster-based framework to mice with HCC increased tumor response to programmed cell death protein 1 (PD-1) immune checkpoint blockade, enhanced cluster of differentiation (CD)4 and CD8 expression, and lowered tumor weight and size (112). 

*Rosmarinic acid*

Rosmarinic acid inhibited angiogenesis and inflammation of hepatocellular carcinoma by reducing tumor growth, lessening the levels of NF-κB p65, IL-1, IL-6, TNF-α, VEGF, TGF-β in comparison to the model group (123). Likewise, rosmarinic acid ameliorated hepatocellular carcinoma by lowering tumor growth, the levels of IL-6, IL-10, signal transducer and activator of transcription 3, Bcl-2, and enhancing the amounts of Bax and caspase-3 (124). On H22 tumor-bearing mice, the combined antitumor effect of rosmarinic acid and indoleamine 2,3-dioxygenase-1 gene silencing was examined concerning the tumor immune microenvironment. Treatment with rosmarinic acid plus indoleamine 2,3-dioxygenase-1-small hairpin RNAs (shRNA) markedly reduced the percentage of splenic Tregs, CD8+ apoptosis, and the levels of IL-10 and TNF-α, while increasing the percentage of CD4+ T cells, the ratio of CD4+/CD8+, and the levels of IL-2 and interferon-γ (IFN-γ). The current investigation showed that rosmarinic acid and indoleamine 2,3-dioxygenase-1-shRNA combination therapy had antitumor effects on HCC (125). Table 2 summarizes the cytotoxic and hepatoprotective properties of *R. officinalis* and its derivative.

**Figure 4:**
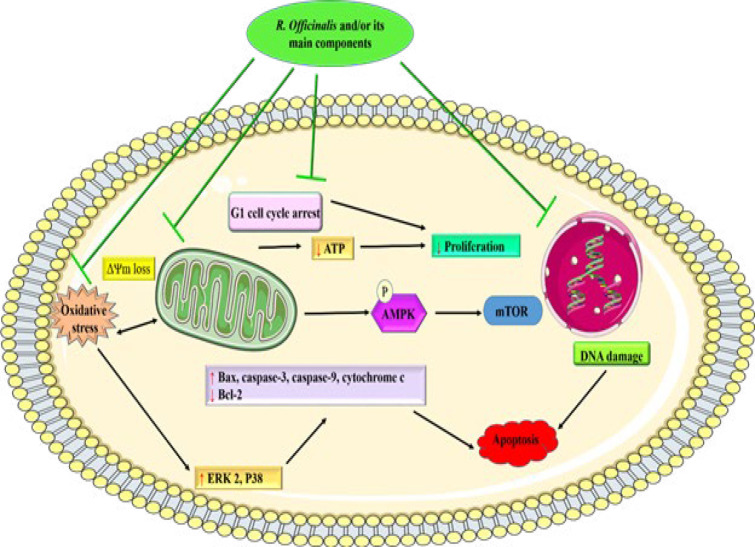
Proposed mechanisms of cytotoxic effects of *Rosmarinus officinalis* and/or its main components on HCC cell lines (images from https://smart.servier.com)

The *in vitro* studies on *R. officinalis* oil and extracts, 1,8-cineole, carnosic acid, carnosol, and rosmarinic acid, have revealed several mechanisms through which these compounds exert their effects on HCC cell lines, including HepG2 cells. These mechanisms include apoptosis, affecting cellular processes, and modulating metabolic pathways in HCC cells. They also increase glucose consumption, activate AMPK and ACC, and decrease mRNA levels of key genes (G6Pase, PGC1α, SIRT1, and LDLR). Moreover, they trigger cell cycle arrest, reduce proliferation, and enhance apoptosis through various pathways, including ROS production and caspase activation. The strengths of these compounds include their potential to induce apoptosis, affect cell cycle regulation, and modulate metabolic pathways in cancer cells (Figure 4). However, a critical appraisal of the included studies reveals several limitations. For instance, methodological details such as experimental conditions are often inadequately reported, which may affect the reproducibility and generalizability of the findings. Additionally, the variability in effectiveness based on different cell lines and experimental setups raises questions about the consistency of these results across various contexts.

In *in vivo* studies, *R. officinalis* extracts, 1,8 cineole, carnosic acid, and rosmarinic acid exhibited promising effects in HCC models. In contrast, carnosic acid administration increased tumor response to immune checkpoint blockade in HCC. Rosmarinic acid has shown potential in inhibiting angiogenesis and inflammation in HCC, reducing tumor growth, and levels of NF-κB p65, IL-1, IL-6, TNF-α, VEGF, and TGF-β. It also ameliorated HCC by lowering tumor growth, IL-6, IL-10, signal transducer and activator of transcription 3, Bcl-2 levels, and enhancing Bax and caspase-3 amounts. 

Despite these promising findings, several research gaps and controversies remain. The incomplete understanding of the biochemical pathways underlying the effects of these compounds necessitates further investigation. Additionally, the variability in study designs and the lack of standardized protocols complicate the interpretation of results and their applicability to clinical settings.

Moreover, the limitations and challenges of using herbal therapies in clinical practice must be addressed. Critical considerations include the quality and purity of herbal products, potential interactions with conventional therapies, and the need for clinical trials to establish safety and efficacy. Future research should focus on elucidating the molecular pathways influenced by these herbal interventions, exploring potential synergistic effects with conventional cancer therapies, and conducting well-designed clinical trials to assess their efficacy and safety in human populations.

### Prospects

This narrative review has shed light on the significant role of the main components of *C. sativus* and *R. officinalis* in managing HCC. Moving forward, various possibilities for future research and development appear, exposing the way for an enhanced understanding and potential therapeutic applications in HCC management.

1. Future studies should focus on understanding the complex molecular pathways by which substances, including crocetin, crocin, safranal, 1,8-cineole, carnosic acid, carnosol, and rosmarinic acid, affect HCC cells. By providing more information about these pathways, we can better understand their exact functions and maximize their therapeutic potential.

2. Exploring potential interactions between these natural substances and conventional cancer treatments is a fascinating field of research. Exploring how these natural chemicals can complement conventional medications may lead to better treatment outcomes and fewer side effects in HCC patients.

3. Transitioning from preclinical investigations to well-designed clinical trials is critical for determining these herbal medications’ safety, efficacy, and optimal dosages in humans. Extensive clinical trials will provide essential information about the real-world application of these compounds in HCC management.

4. Considering the potential variability in effectiveness based on cell lines and experimental conditions, personalized medicine techniques could assist in customizing treatment regimens for individual patients, maximizing therapeutic advantages while minimizing adverse effects.

5. Investigating the combination of *C. sativus* and *R. officinalis *components with additional medications or treatment methods could provide innovative combination therapies for HCC management.

6. Developing advanced formulations for these substances, such as nanotechnology-based delivery systems, liposomal formulations, solid dispersions, hydrogels, and polymeric nanoparticles, can improve their bioavailability, stability, and target delivery. These formulations might have the potential to improve the therapeutic efficacy of *C. sativus* and *R. officinalis* and their main components used in HCC treatment.

## Conclusion

The evidence provided in this narrative review indicates that the main components of *C. sativus* and *R. officinalis* significantly affect HCC management. Crocetin, crocin, safranal, 1,8-cineole, carnosic acid, carnosol, and rosmarinic acid have all been shown to effectively target HCC cells by various methods *in vitro* and *in vivo*.

**Figure 5:**
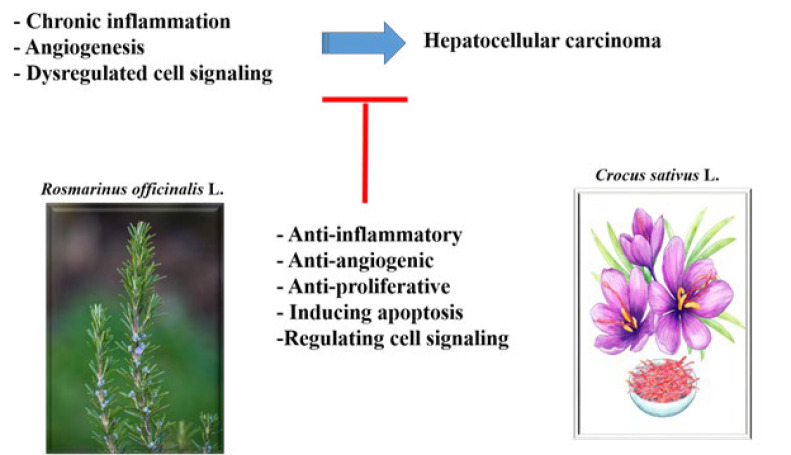
Ameliorative effects of *Crocus sativus* and *Rosmarinus officinalis* on HCC (images from https://smart.servier.com and https://www.freepik.com/)

*In vitro* investigations have revealed that these substances exhibit cytotoxic effects, induce apoptosis, and modulate important signaling pathways, highlighting their therapeutic potential. Notably, *C. sativus* extracts and its main components have shown the potential to inhibit liver cancer progression by modulating proliferation, apoptosis, and inflammatory pathways (Figure 5).

Furthermore, studies on *R. officinalis* main components demonstrated various mechanisms of action in HCC cell lines, exhibiting the ability to influence apoptosis, cellular processes, and metabolic pathways. *In vivo* investigations have shown the promise of these compounds in HCC models, with implications for improving immune responses while reducing angiogenesis and inflammation in the tumor microenvironment.

While these findings are remarkable, gaps persist in our understanding of the underlying biochemical processes and potential variations in efficacy across different experimental conditions. Further study is needed to fill these information gaps, investigate synergies with conventional cancer treatments, and progress toward clinical studies to assess the safety and efficacy of these herbal medicines in humans. By thoroughly examining the molecular pathways affected by these natural compounds and conducting clinical trials, their full therapeutic potential in treating HCC could be realized.

HCC: Hepatocellular carcinoma

HCC: Hepatocellular carcinoma

HCC: Hepatocellular carcinoma

**Table 1:** Effect of *Crocus sativus* on HCC

Compound	Study design	Doses/Duration	Results	Ref.
*In vitro*				
*C. sativus* extract	HepG-2 cells	50-400 μg/ml	↓ Colony formation	(126)
*C. sativus* aqueous extract	HepG-2 cells	0, 200, 400, 800 μg/ml, 6-72 hr	↑ Cytotoxic effect ↓ NO concentration	(65)
*C. sativus* aqueous-ethanolic extract	HepG2 cells	1.0, 2.0, 4.0, 6.0 mg/ml, 6, 24, 48 hr	↑ Caspase-3 cleavage, DNA damage, cell cycle arrest *↓*NF-κB activation, enhance caspase-3 cleavage, cause DNA damage	(66)
*C. sativus*	QGY-7703 cells	-	- Stopped the cell cycle at the G0/G1 phase ↑ Apoptosis, senescent cells, Bax/Bcl-2 ratio, P21 expression ↓Cell growth, hTERT levels	(67)
Crocetin	PLC/PRF5 and Hep3B cells	50 μM, 6,12, 24 hr	↑ Apoptosis, SHP-1 expression ↓ Invasive potential of HCC cells, cell proliferation, constitutive/inducible STAT3 activation, STAT3 nuclear accumulation, its DNA binding activity in HCC cells, activity of JAK1, JAK2, and Src, Bcl-2, Bcl-xL, cyclin D1, survivin, VEGF, COX-2, MMP-9	(68)
Crocetin- poly (ethylene glycol)-magnetic nanoparticles	HepG-2 cells	0.05, 0.5, 3, 5 mg/ml, 24, 72 hr	↓ Cell proliferation	(69)
Crocin	HepG-2 cells	3 mg/ml, 48 hr	↑ Cytotoxicity ↓ Telomerase activity, expression level of the catalytic subunit of the hTERT gene	(70)
Crocin	HepG-2 cells	0.01, 0.03, 0.1, 0.3, 1 mM, 24, 48 hr	- Arrested the cell cycle at specific phases ↑ Apoptosis ↓ Inflammation	(71)
Crocin-coated magnetite nanoparticles	HepG-2 cells	0.05, 0.07, 0.09, 0.1 mg Fe/ml, 72 hr	↓ Cell Proliferation	(72)
Crocin	HepG2 and HCCLM3 cells	3 mg/ml, 6 hr	↑ LC3 puncta, LC3-II accumulation ↓ Akt/mTOR signaling pathway	(73)
Crocin	Hep3B and HepG2 cells	20 μM, 0–24 hr	↑ SHP-1 production, apoptosis, Bax ↓IL-6-induced STAT3 activation, STAT3 phosphorylation, VEGF, CXCR-4, cyclin D1, Bcl-2, survivin	(74)
Crocin	HepG-2 cells	100, 150, 200, 250, 300 μM, 48 hr	↑ Antitumor effect	(75)
Crocin nanoparticles	HepG-2 cells	-	↑ Antitumor activity, apoptosis, cell cycle arrest at the G2/M phase. Apoptotic and autophagic genes	(76)
Safranal	HepG-2 cells	50, 100, 500, 700, 900 μM, 24, 48, 72 hr	↑Cell cycle arrest in the G2/M phase at 6 and 12 h and the S-phase at 24 h, pro-apoptotic effects	(77)
Safranal	HepG-2 cells	300, 500, 700 μM, 24 hr	↓VEGF secretion, expression of p-AKT, p-ERK1/2, MMP9, p-FAK, p-STAT3, HIF-1α, VEGF, VEGFR2	(78)
Safranal	HepG-2 cells	0.01, 0.03, 0.1, 0.3, 1 mM, 24, 48 hr	↑ Caspase-3 and -7 activities ↓ Cell viability, IL-8 secretion	(79)
Safranal	HepG-2 cells	500 uM, 24 hr	↑ DNA damage, protein destabilization, oxidative stress	(80)
*In vivo*				
*C. sativus* aqueous-ethanolic extract	Male rats	75, 150, 300 mg/kg, 22 weeks, PO	↑ SOD, CAT, glutathione-S-transferase levels ↓ Placental glutathione S-transferase-positive foci, hepatic dyschromatic nodules, myeloperoxidase activity, MDA, and protein carbonyl production in the liver, oxidative stress, NF-κB p-65, COX-2, iNOS, Ki-67, phosphorylated TNF receptor	(66)
*C. sativus* aqueous extract	Male Sprague-Dawley rats	300 mg /kg, 8 weeks, gavage	↑ Albumin levels, total antioxidant capacity levels ↓ Hepatic dyschromatic nodules, TGF-β levels, ALT, and AST) activities	(81)
*C. sativus* aqueous extract	Male Sprague-Dawley rats	300 mg /kg, 8 weeks, gavage	↑ Serum CAT, GSH, SOD, active caspase 3 expression, ↓ MMP-9 production, incidence and number of hepatic dyschromatic nodules	(82)
*Crocus sativus* silver nanoparticles	Male albino rats	200 mg/kg, 6 weeks, IP	↓Carcinogenesis, atypical cells	(83)
Crocin	Male albino Wistar rats	100, 200 mg/kg, 12 weeks, PO	↑ Anti-proliferative, pro-apoptotic, anti-inflammatory properties by ↓NF-kB	(71)
Crocin-coated magnetite nanoparticles	Male Balb/c mice	11, 22 mg/kg, 2 weeks, IV	↑ Apoptotic cells ↓ Precancerous lesions, indicators linked to angiogenesis, inflammation, oxidative stress, and cell proliferation	(72)
Crocin	Male Sprague-Dawley rats	10 mg/kg, 4 weeks, IP	↑ HO-1 and Nrf2 expression in the liver, antioxidant defenses, expression of p53 gene and TRAIL, apoptosis ↓ Malignant lesions Keap-1 expression, c-JNK levels	(84)
Crocin	Male albino Wistar rats	50 mg/kg, 6 weeks, gavage	↓ Weight loss, CRP, IL-6, LDH, oxidative stress markers, histopathological alterations, NF-κB p53, TNF-α, VEGF	(75)
Crocin	Albino Wistar rats	200 mg/kg, 3 weeks, PO	↓ Dyschromatic nodules, dysplastic nodules, nodule multiplicity, proliferative cell and β-catenin levels in tumor tissues, COX-2 and NF-κB	(85)

Akt: protein kinase B; ALT: alanine aminotransferase; AST: aspartate aminotransferase; Bax: Bcl-2-associated X protein; Bcl-2: B-cell lymphoma 2; Bcl-xL: B-cell lymphoma-extra-large; CAT: catalase; c-JNK: c-Jun N-terminal kinase; COX-2: cyclooxygenase-2; CRP: C-reactive protein; CXCR-4: C-X-C chemokine receptor type 4; DNA: deoxyribonucleic acid; ERK: extracellular signal-regulated kinase; FAK: focal adhesion kinase; GSH: glutathione; HCC: hepatocellular carcinoma; HIF-1α: hypoxia-inducible factor 1-alpha; HO-1: heme oxygenase-1; hTERT: human telomerase reverse transcriptase; IL: interleukin; iNOS: inducible nitric oxide synthase; JAK: Janus kinase; LC3: microtubule-associated protein 1A/1B-light chain 3; LDH: lactate dehydrogenase; MDA: malondialdehyde; MMP-9: matrix metalloproteinase-9; mTOR: mammalian target of rapamycin; NF-κB: nuclear factor-kappa B; NO: nitric oxide; Nrf2: nuclear factor erythroid 2-related factor 2; Ref: references; SHP-1: Src homology region 2 domain-containing phosphatase-1; SOD: superoxide dismutase; STAT3: signal transducer and activator of transcription 3; TGF-β: transforming growth factor Beta; TNF-α: tumor necrosis factor alpha; TRAIL: TNF-related apoptosis-inducing ligand; VEGF: vascular endothelial growth factor; VEGFR2: vascular endothelial growth factor receptor 2

**Table 2:** Effect of *Rosmarinus officinalis* on HCC

Compound	Study design	Doses/Duration	Results		Ref.
*In vitro*					
*R. officinalis* oils	Hep-G2 cells	-	↑ Bax expression ↓ Bcl-2 expression		(100)
*R. officinalis* oil	Hep-G2 cells	7.5–93.75×10–3‰, 24 hr	↑ Cytoplasmic membrane shrinkage, apoptotic bodies development, internucleosomal DNA fragmentation, DNA ladder formation, cell accumulation in the G1 phase, cell death ↓ Number of cells in the S phase		(101)
*R. officinalis* methanolic extract	Hep-G2 cells	2, 10, 50 μg/ml	↑Glucose consumption, phosphorylation of AMPK and ACC ↓ mRNA level of G6Pase, PGC1α SIRT1, and LDLR, expression of ACCB		(102)
*R. officinalis* extract	Hep-G2 cells	0, 10, 20, 30, 40, 50, 75, 100 μg/ml	↑ Sestrin2 and MRP2 expression, Nrf2 protein level		(103)
1,8 cineole	HepG2 cells	0-10 mM, 24-72 h	↑ G0/G1 arrest, cellular senescence, ROS production, p38, ERK, and AMPK activation ↓ Cell proliferation, mTOR		(104)
Carnosic acid	Hep-G2 cells	10 μM, 24 hr	↑Phosphorylation of MAPK and EGFR ↓PPARγ level		(105)
Carnosic acid	Hep-G2 cells	2.5-10 μmol/l, 6 hr	↑ Cell viability, GSH, MnSOD, Bcl-xL, SIRT1 ↓ LDH activity, caspase-3		(106)
Carnosic acid	Hep-G2 cells	0–100 μM, 4–24 hr	↑ Rapid caspase-3 activation, proteolytic cleavage of PARP, ↓ Cell viability, Bcl-2/Bax protein, phosphorylation of Akt		(107)
Carnosic acid	HepG2, SMMC-7721 cells	30–150 μM, 24 hr	↑ Apoptosis, caspase-3, caspase-8, caspase-9 activities, intracellular ROS ↓ Cell viability, proliferation, migration, mitochondrial membrane potential		(103)
Carnosic acid	Hep-G2 cells	0, 10, 20, 30, 40, 50, 75, 100 μM, 24–72 hr	↑ Sestrin2 and MRP2 expression, Nrf2 protein level		(103)
Carnosic acid	Huh7, HepG2 cells	10 μM	↑ Sorafenib-induced cell death in the neoplastic cell line, cytotoxic autophagy, apoptosis, DNA damage, ROS, cell cycle prolongation ↓ HCC cells proliferation and survival, cell growth, activation of STAT3 transcription factor and ERK1/2		(109)
Carnosic acid	Huh7 (Sorafenib-sensitive), HCO2 (Sorafenib–resistant)	10 μM	↑ HCC cell death, autophagy, apoptosis, perinuclear aggregation of LC3 cytoplasmic vacuolation, protein levels of Atg3, Beclin1, and LC3		(110)
Carnosic acid	HepG2 cells	0-30 μM	↑ Phosphorylation of AMPK and ACC1 ↓ G6PC, PCK1, mRNA levels of ACC1, FAS, and SREBP-1c, cell proliferation, cell viability		(111)
Carnosic acid nanocluster-based framework	MHCC97H cells	-	↑ Apoptosis ↓ Migration, invasion, proliferation, Wnt/β-catenin signaling pathway activity		(112)
Carnosol	HepG2 cells	0, 10, 20, 30, 40, 50, 75, 100 μM, 24–72 hr		↑ Sestrin2 and MRP2 expression, Nrf2 protein level	(103)
Carnosol	HepG2 cells	0–30 μM		↑ Phosphorylation of AMPK and ACC1 ↓G6PC, PCK1, mRNA levels of ACC1, FAS, and SREBP-1c, cell proliferation, cell viability	(111)
Carnosol	HepG2, Huh7 cells	-		↑ Apoptosis, AMPK-p53 pathway activity ↓ Cell viability, colony formation, effort, invasion, migration	(113)
Rosmarinic acid	HepG2 cells	6.25, 12.5, 25, 50, 100 μg/ml, 24-72 hr		↑ Apoptosis, cell cycle arrest in G1 ↓Cell viability, cell growth, production of lactate and ATP, glucose uptake, expression of hexokinase-2 and glucose transporter-1	(114)
Rosmarinic acid	HepG2, Bel-7402 Cells	25, 50, 100 μg/ml, 12–48 hr	↑ Damaged cell morphology, apoptosis, DNA damage ↓ Cell viability, S-phase cell numbers, Bcl-2 expression, Bax expression		(115)
Rosmarinic acid	SMMC 7721 cells	0, 20, 50, 100 μmol/l, 24–72 hr	↑ G1 arrest, apoptosis ↓ Cell proliferation, invasion		(116)
Rosmarinic acid	HepG2 cells	1, 10 μM, 24 hr	↑ Nrf2-Keap1 antioxidant pathway activity		(127)
Rosmarinic acid	HepG2 cells	12.5, 25, 50, 100, 200 μg/ml, 24 hr	↑ Antioxidant property, dose-dependent cell death, apoptosis, cell cycle arrest at Go/G1 phase, Bax, ERK2 ↓ Bcl-2 mRNA expression		(117)
Rosmarinic acid	HepG2 cells	10, 100, 1000 µM, 24 hr	↑ Levels of polyubiquitinated protein, LC3B-II, HSP70, BiP, ATF4, protein carbonyl, and cleaved PARP1 ↓ Cell viability		(118)
Rosmarinic acid	HepG2 cells	0, 2.5, 5, 10, 20, 40, 80, 160, 320 μM	↑ Apoptosis, Bax, caspase-3, caspase-9, ↓ Cell viability, Bcl-2, cell migration, invasion		(119)
Rosmarinic acid	HepG2 cells	50, 75, 100 μg/ml, 48 hr	↑ Apoptosis rate, cleaved caspase-3, cytochrome C, Bax expression cell cycle arrest in the S phase ↓ Cell viability, mobility, Bcl-2 expression		(120)
*In vivo*					
*R. officinalis* ethanolic extract	Male albino rats	200 mg/kg, 4 weeks, PO	↑ Total protein, albumin, ↓ ALT, AST, ALP, GGT, LDH, AFP		(121)
1,8 cineole	Rats	100 mg/kg, 4 weeks, PO	↑ Functional capacity, hepatic miR-122 level ↓Ferritin, alpha-L-fucosidase, arginase-1, alpha-fetoprotein, EMT, FSCN1, MMP-9, TGF-β1, VEGF, vimentin		(122)
Carnosic acid nanocluster-based framework	BALB/C nude mice	-	↑ Tumor response to PD-1 immune checkpoint blockade, CD4 and CD8 expression ↓ Tumor weight and size		(112)
Rosmarinic acid	SPF male Kunming mice	75, 150, 300 mg/kg, 10 days, IG	↓ Tumor growth, NF-kB p65, IL-1, IL-6, TNF-α, VEGF, TGF-β		(123)
Rosmarinic acid	SPF male Kunming mice	75, 150, and 300 mg/kg, 10 days, gavage	↑ Bax, caspase-3 ↓ Tumor growth, IL-6, IL-10, and signal transducer and activator of transcription 3, Bcl-2		(124)
Rosmarinic acid	H22 tumor-bearing mice	-	↑ CD4+ T cells, CD4+/CD8+ ratio, IL-2, IFN-γ ↓ CD8+ apoptosis, the proportion of splenic Tregs proportion, IL-10, TNF-α		(125)

ACC: acetyl-CoA carboxylase; AFP: alpha-fetoprotein; Akt: protein kinase B; ALP: alkaline phosphatase; ALT: alanine aminotransferase; AMPK: AMP-activated protein kinase; AST: aspartate aminotransferase; Atg3: autophagy-related protein 3; Bax: Bcl-2-associated X protein; Bcl-2: B-cell lymphoma 2; CD: cluster of differentiation; EGFR: epidermal growth factor receptor; EMT: epithelial-mesenchymal transition; ERK: extracellular signal-regulated kinase; FAS: fatty acid synthase; FSCN1: Fascin actin-bundling protein 1; G6Pase: glucose-6-phosphatase; G6PC: glucose-6-phosphatase catalytic subunit; GGT: gamma-glutamyl transferase; GSH: glutathione; HCC: hepatocellular carcinoma; i.g.: intragastric; IFN-γ: interferon-gamma; IL: interleukin; LC3: microtubule-associated protein 1A/1B-light chain 3; LDH: lactate dehydrogenase; LDLR: low-density lipoprotein receptor; MAPK: mitogen-activated protein kinase; miR: microRNA; MMP-9: matrix metallopeptidase 9; MnSOD: manganese superoxide dismutase; mTOR: mammalian target of rapamycin; NF-kB: nuclear factor kappa-light-chain-enhancer of activated B cells; Nrf2: nuclear factor erythroid 2-related factor 2; p.o.: Per os (Orally); PARP: poly (ADP-ribose) polymerase; PCK1: phosphoenolpyruvate carboxykinase 1; PGC1α: peroxisome proliferator-activated receptor gamma coactivator 1-alpha; PPARγ: peroxisome proliferator-activated receptor gamma; ref: reference; ROS: reactive oxygen species; SIRT1: Sirtuin 1; SREBP-1c: sterol regulatory element-binding protein 1c; STAT3: signal transducer and activator of transcription 3; TGF-β: transforming growth factor beta; TNF-α: tumor necrosis factor alpha; VEGF: vascular endothelial growth factor; Wnt: wingless/integrated.

HCC: Hepatocellular carcinoma

HCC: Hepatocellular carcinoma

## Data Availability

Data sharing does not apply to this article as no datasets were generated or analyzed during the current study.

## Declaration

During the preparation of this work**,** the author(s) used ChatGPT and Quillbot to rephrase to reduce plagiarism and improve the language and grammar. After using this tool/service, the author(s) reviewed and edited the content as needed and take(s) full responsibility for the published content.

## References

[1] Llovet JM, Zucman-Rossi J, Pikarsky E, Sangro B, Schwartz M, Sherman M (2016). Hepatocellular carcinoma. Nat Rev Dis Primers.

[2] Villanueva A (2019). Hepatocellular carcinoma. N Engl J Med.

[3] Llovet JM, Kelley RK, Villanueva A, Singal AG, Pikarsky E, Roayaie S (2021). Hepatocellular carcinoma. Nat Rev Dis Primers.

[4] Akinyemiju T, Abera S, Ahmed M, Alam N, Alemayohu MA, Allen C (2017). The burden of primary liver cancer and underlying etiologies from 1990 to 2015 at the global, regional, and national level: Results from the global burden of disease study 2015. JAMA Oncol.

[5] Kanwal F, Kramer J, Asch SM, Chayanupatkul M, Cao Y, El-Serag HB (2017). Risk of hepatocellular cancer in HCV patients treated with direct-acting antiviral agents. Gastroenterology.

[6] Estes C, Razavi H, Loomba R, Younossi Z, Sanyal AJ (2018). Modeling the epidemic of nonalcoholic fatty liver disease demonstrates an exponential increase in burden of disease. Hepatology.

[7] Schulze K, Imbeaud S, Letouzé E, Alexandrov LB, Calderaro J, Rebouissou S (2015). Exome sequencing of hepatocellular carcinomas identifies new mutational signatures and potential therapeutic targets. Nat Genet.

[8] Ogunwobi OO, Harricharran T, Huaman J, Galuza A, Odumuwagun O, Tan Y (2019). Mechanisms of hepatocellular carcinoma progression. World J Gastroenterol.

[9] Siegel RL, Miller KD, Jemal A (2018). Cancer statistics, 2018. CA Cancer J Clin.

[10] Dimitroulis D, Damaskos C, Valsami S, Davakis S, Garmpis N, Spartalis E (2017). From diagnosis to treatment of hepatocellular carcinoma: An epidemic problem for both developed and developing world. World J Gastroenterol.

[11] Daher S, Massarwa M, Benson AA, Khoury T (2018). Current and future treatment of hepatocellular carcinoma: An updated comprehensive review. J Clin Transl Hepatol.

[12] Golabi P, Fazel S, Otgonsuren M, Sayiner M, Locklear CT, Younossi ZM (2017). Mortality assessment of patients with hepatocellular carcinoma according to underlying disease and treatment modalities. Medicine.

[13] Ikeda M, Morizane C, Ueno M, Okusaka T, Ishii H, Furuse J (2018). Chemotherapy for hepatocellular carcinoma: current status and future perspectives. Jpn J Clin Oncol.

[14] Keating GM (2017). Sorafenib: A review in hepatocellular carcinoma. Target Oncol.

[15] Personeni N, Pressiani T, Santoro A, Rimassa L (2018). Regorafenib in hepatocellular carcinoma: Latest evidence and clinical implications. Drugs Context.

[16] Spallanzani A, Orsi G, Andrikou K, Gelsomino F, Rimini M, Riggi L (2018). Lenvatinib as a therapy for unresectable hepatocellular carcinoma. Expert Rev Anticancer Ther.

[17] Hosseini M, Pkan P, Rakhshandeh H, Aghaie A, Sadeghnia HR, Rahbardar MG (2011). The effect of hydro-alcoholic extract of citrus flower on pentylenetetrazole and maximal electroshock-induced seizures in mice. World Appl Sci J.

[18] Jalali J, Ghasemzadeh Rahbardar M (2023). Ameliorative effects of Portulaca oleracea L (purslane) and its active constituents on nervous system disorders: A review. Iran J Basic Med Sci.

[19] Sharma AN, Dewangan HK, Upadhyay PK (2024). Comprehensive review on herbal medicine: emphasis on current therapy and role of phytoconstituents for cancer treatment. Chem Biodivers.

[20] Ali M, Wani SUD, Salahuddin M, Manjula S, Mruthunjaya K, Dey T (2023). Recent advance of herbal medicines in cancer-a molecular approach. Heliyon.

[21] Park MN, Rahman MA, Rahman MH, Kim JW, Choi M, Kim JW (2022). Potential therapeutic implication of herbal medicine in mitochondria-mediated oxidative stress-related liver diseases. Antioxidants.

[22] Fadishei M, Ghasemzadeh Rahbardar M, Imenshahidi M, Mohajeri A, Razavi BM, Hosseinzadeh H (2021). Effects of Nigella sativa oil and thymoquinone against bisphenol A-induced metabolic disorder in rats. Phytother Res.

[23] Emadi SA, Ghasemzadeh Rahbardar M, Mehri S, Hosseinzadeh H (2022). A review of therapeutic potentials of milk thistle (Silybum marianum L ) and its main constituent, silymarin, on cancer, and their related patents. Iran J Basic Med Sci.

[24] Mohammadi Zonouz A, Ghasemzadeh Rahbardar M, Hosseinzadeh H (2024). The molecular mechanisms of ginkgo (Ginkgo biloba) activity in signaling pathways: A comprehensive review. Phytomedicine.

[25] Ghasemzadeh Rahbardar M, Hosseinzadeh H (2024). The ameliorative effect of turmeric (Curcuma longa Linn) extract and its major constituent, curcumin, and its analogs on ethanol toxicity. Phytother Res.

[26] Ardakanian A, Ghasemzadeh Rahbardar M, Omidkhoda F, Razavi BM, Hosseinzadeh H (2022). Effect of alpha-mangostin on olanzapine-induced metabolic disorders in rats. Iran J Basic Med Sci.

[27] Salehsari A, Ghasemzadeh Rahbardar M, Razavi BM, Hosseinzadeh H (2024). Investigating the effect of zeaxanthin on olanzapine-induced metabolic disorders in rats. Avicenna J Phytomed.

[28] Panneerselvam S, Wilson C, Kumar P, Abirami D, Pamarthi J, Reddy MS (2023). Overview of hepatocellular carcinoma: from molecular aspects to future therapeutic options. Cell Adh Migr.

[29] Liao X, Bu Y, Jia Q (2020). Traditional Chinese medicine as supportive care for the management of liver cancer: Past, present, and future. Genes Dis.

[30] Li M, Liu Y, Zhang H, Liu Y, Wang W, You S (2022). Anti-cancer potential of polysaccharide extracted from Polygonatum sibiricum on HepG2 cells via cell cycle arrest and apoptosis. Front Nutr.

[31] Kaewnoonual N, Itharat A, Pongsawat S, Nilbu-Nga C, Kerdput V, Pradidarcheep W (2020). Anti-angiogenic and anti-proliferative effects of Benja-ummarit extract in rats with hepatocellular carcinoma. Biomed Rep.

[32] Sahu N, Rakshit S, Bhaskar LVKS, Nagaraju GP, Peela S (2022). Chapter 3 - Risk factors and pathogenic mechanism–associated hepatocellular carcinoma. Theranostics and Precision Medicine for the Management of Hepatocellular Carcinoma.

[33] Seung KY (2018). Molecular mechanism of hepatocellular carcinoma. Hepatoma Res.

[34] Li Y, Yu Y, Yang L, Wang R (2023). Insights into the role of oxidative stress in hepatocellular carcinoma development. Front Biosci (Landmark Ed).

[35] Refolo MG, Messa C, Guerra V, Carr BI, D’Alessandro R (2020). Inflammatory mechanisms of HCC development. Cancers.

[36] Borgia M, Dal Bo M, Toffoli G (2021). Role of virus-related chronic inflammation and mechanisms of cancer immune-suppression in pathogenesis and progression of hepatocellular carcinoma. Cancers.

[37] Bishayee A (2014). The role of inflammation and liver cancer. Adv Exp Med Biol.

[38] Villanueva A, Luedde T (2016). The transition from inflammation to cancer in the liver. Clin Liver Dis (Hoboken).

[39] Alqahtani A, Khan Z, Alloghbi A S, Said Ahmed T, Ashraf M M, Hammouda D (2019). Hepatocellular carcinoma: Molecular mechanisms and targeted therapies. Medicina.

[40] Ramakrishna G, Rastogi A, Trehanpati N, Sen B, Khosla R, Sarin SK (2013). From cirrhosis to hepatocellular carcinoma: New molecular insights on inflammation and cellular senescence. Liver Cancer.

[41] Luedde T, Schwabe RF (2011). NF-κB in the liver--linking injury, fibrosis and hepatocellular carcinoma. Nat Rev Gastroenterol Hepatol.

[42] Soukupova J, Malfettone A, Hyroššová P, Hernández-Alvarez MI, Peñuelas-Haro I, Bertran E (2017). Role of the transforming growth factor-β in regulating hepatocellular carcinoma oxidative metabolism. Sci Rep.

[43] Aravalli RN, Steer CJ, Cressman EN (2008). Molecular mechanisms of hepatocellular carcinoma. Hepatology.

[44] Waly Raphael S, Yangde Z, Yuxiang C (2012). Hepatocellular carcinoma: Focus on different aspects of management. ISRN Oncol.

[45] Chen C, Wang G (2015). Mechanisms of hepatocellular carcinoma and challenges and opportunities for molecular targeted therapy. World J Hepatol.

[46] Bertino G, Demma S, Ardiri A, Proiti M, Gruttadauria S, Toro A (2014). Hepatocellular carcinoma: Novel molecular targets in carcinogenesis for future therapies. Biomed Res Int.

[47] Cha C, Dematteo RP (2005). Molecular mechanisms in hepatocellular carcinoma development. Best Pract Res Clin Gastroenterol.

[48] Kudo M (2011). Signaling pathway and molecular-targeted therapy for hepatocellular carcinoma. Dig Dis.

[49] Roberts LR, Gores GJ (2005). Hepatocellular carcinoma: Molecular pathways and new therapeutic targets. Semin Liver Dis.

[50] Dawson SP (2008). Hepatocellular carcinoma and the ubiquitin-proteasome system. Biochim Biophys Acta.

[51] Zolfaghari Farajerdi M, Rajabian F, Razavi BM, Ghasemzadeh Rahbardar M, Khajavi Rad A, Amoueian S (2025). Evaluating the effect of crocin on contrast-induced nephropathy in rats. Avicenna J Phytomed.

[52] Javadi B, Sahebkar A, Emami SA (2013). A survey on saffron in major islamic traditional medicine books. Iran J Basic Med Sci.

[53] Jiang H, Huang X, Wang J, Zhou Y, Ren C, Zhou T (2023). Hepatoprotective effect of medicine food homology flower saffron against CCl(4)-induced liver fibrosis in mice via the Akt/HIF-1α/VEGF signaling pathway. Molecules.

[54] Ghasemzadeh Rahbardar M, Hosseinzadeh H (2023). A review of how the saffron (Crocus sativus) petal and its main constituents interact with the Nrf2 and NF-κB signaling pathways. Naunyn Schmiedebergs Arch Pharmacol.

[55] Naraki K, Ghasemzadeh Rahbardar M, Razavi BM, Aminifar T, Khajavi Rad A, Amoueian S (2024). The power of trans-sodium crocetinate: exploring its renoprotective effects in a rat model of colistin-induced nephrotoxicity. Naunyn Schmiedebergs Arch Pharmacol.

[56] Rajabian F, Mehri S, Razavi BM, Khajavirad A, Ghasemzadeh Rahbardar M, Hosseinzadeh H (2023). Effect of trans-sodium crocetinate on contrast-induced cytotoxicity in HEK-293 cells. Iran J Basic Med Sci.

[57] Vafaeipour Z, Ghasemzadeh Rahbardar M, Hosseinzadeh H (2023). Effect of saffron, black seed, and their main constituents on inflammatory cytokine response (mainly TNF-α) and oxidative stress status: an aspect on pharmacological insights. Naunyn Schmiedebergs Arch Pharmacol.

[58] Ghasemzadeh Rahbardar M, Ferns GA, Ghayour Mobarhan M (2025). Assessing the efficacy of herbal supplements for managing obesity: A comprehensive review of global clinical trials. Iran J Basic Med Sci.

[59] Aminifard T, Mehri S, Ghasemzadeh Rahbardar M, Rajabian F, Khajavi Rad A, Hosseinzadeh H (2024). Trans-sodium crocetinate suppresses apoptotic and oxidative response following myoglobin-induced cytotoxicity in HEK-293 cells. Iran J Basic Med Sci.

[60] Boskabady MH, Ghasemzadeh Rahbardar M, Nemati H, Esmaeilzadeh M (2010). Inhibitory effect of Crocus sativus (saffron) on histamine (H1) receptors of guinea pig tracheal chains. Pharmazie.

[61] Boskabady MH, Rahbardar MG, Jafari Z (2011). The effect of safranal on histamine (H(1)) receptors of guinea pig tracheal chains. Fitoterapia.

[62] Rajabalizadeh R, Ghasemzadeh Rahbardar M, Razavi BM, Hosseinzadeh H (2024). Renoprotective effects of crocin against colistin-induced nephrotoxicity in a rat model. Iran J Basic Med Sci.

[63] Rajabian F, Razavi BM, Mehri S, Amouian S, Ghasemzadeh Rahbardar M, Khajavi Rad A (2025). Evaluation of pathways involved in the protective effect of trans sodium crocetinate against contrast-induced nephropathy in rats. Naunyn Schmiedebergs Arch Pharmacol.

[64] Mohammadzadeh L, Ghasemzadeh Rahbardar M, Razavi BM, Hosseinzadeh H (2022). Crocin protects malathion-induced striatal biochemical deficits by inhibiting apoptosis and increasing α-synuclein in rats’ striatum. J Mol Neurosci.

[65] Parizadeh MR, Ghafoori Gharib F, Abbaspour AR, Tavakol Afshar J, Ghayour - Mobarhan M (2011). Effects of aqueous saffron extract on nitric oxide production by two human carcinoma cell lines: Hepatocellular carcinoma (HepG2) and laryngeal carcinoma (Hep2). Avicenna J Phytomed.

[66] Amin A, Hamza AA, Bajbouj K, Ashraf SS, Daoud S (2011). Saffron: A potential candidate for a novel anticancer drug against hepatocellular carcinoma. Hepatology.

[67] Liu T, Tian L, Fu X, Wei L, Li J, Wang T (2020). Saffron inhibits the proliferation of hepatocellular carcinoma via inducing cell apoptosis. Panminerva Med.

[68] Mohan CD, Kim C, Siveen KS, Manu KA, Rangappa S, Chinnathambi A (2021). Crocetin imparts antiproliferative activity via inhibiting STAT3 signaling in hepatocellular carcinoma. IUBMB Life.

[69] Ibrahim S, Baig B, Hisaindee S, Darwish H, Abdel-Ghany A, El-Maghraby H (2023). Development and evaluation of crocetin-functionalized pegylated magnetite nanoparticles for hepatocellular carcinoma. Molecules.

[70] Noureini SK, Wink M (2012). Antiproliferative effects of crocin in HepG2 cells by telomerase inhibition and hTERT down-regulation. Asian Pac J Cancer Prev.

[71] Amin A, Hamza AA, Daoud S, Khazanehdari K, Hrout AA, Baig B (2016). Saffron-based crocin prevents early lesions of liver cancer: In vivo, in vitro and network analyses. Recent Pat Anticancer Drug Discov.

[72] El-Kharrag R, Amin A, Hisaindee S, Greish Y, Karam SM (2017). Development of a therapeutic model of precancerous liver using crocin-coated magnetite nanoparticles. Int J Oncol.

[73] Yao C, Liu BB, Qian XD, Li LQ, Cao HB, Guo QS (2018). Crocin induces autophagic apoptosis in hepatocellular carcinoma by inhibiting Akt/mTOR activity. Onco Targets Ther.

[74] Kim B, Park B (2018). Saffron carotenoids inhibit STAT3 activation and promote apoptotic progression in IL-6-stimulated liver cancer cells. Oncol Rep.

[75] Abdu S, Juaid N, Amin A, Moulay M, Miled N (2022). Therapeutic effects of crocin alone or in combination with sorafenib against hepatocellular carcinoma: In vivo & in vitro insights. Antioxidants.

[76] Basuony NS, Mohamed TM, Beltagy DM, Massoud AA, Elwan MM (2025). Therapeutic effects of crocin nanoparticles alone or in combination with doxorubicin against hepatocellular carcinoma in vitro. Anticancer Agents Med Chem.

[77] Al-Hrout Aa, Chaiboonchoe A, Khraiwesh B, Murali C, Baig B, El-Awady R (2018). Safranal induces DNA double-strand breakage and ER-stress-mediated cell death in hepatocellular carcinoma cells. Sci Rep.

[78] Abdalla A, Murali C, Amin A (2022). Safranal inhibits angiogenesis via targeting HIF-1α/VEGF machinery: In vitro and ex vivo insights. Front Oncol.

[79] Abdalla Y, Abdalla A, Hamza AA, Amin A (2021). Safranal prevents liver cancer through inhibiting oxidative stress and alleviating inflammation. Front Pharmacol.

[80] Nelson DR, Hrout AaA, Alzahmi AS, Chaiboonchoe A, Amin A, Salehi-Ashtiani K (2022). Molecular mechanisms behind safranal’s toxicity to HepG2 cells from dual omics. Antioxidants.

[81] Zein N (2017). The effect of Saffron aqueous extract on hepatocellular carcinoma rat model. Biochem Lett.

[82] Fikry R, Zein N, Faozan A (2018). Properties of Crocus sativus saffron on DEN-induced hepatocellular carcinoma in rats. Biochem Lett.

[83] Thamer NA (2016). Green synthesized silver nanoparticles using Crocus sativus L extract after reduces prehepatocellular carcinoma in rats. Iraqi J Cancer Med Genet.

[84] Elsherbiny NM, Eisa NH, El-Sherbiny M, Said E (2020). Chemo-preventive effect of crocin against experimentally-induced hepatocarcinogenesis via regulation of apoptotic and Nrf2 signaling pathways. Environ Toxicol Pharmacol.

[85] Awad B, Hamza AA, Al-Maktoum A, Al-Salam S, Amin A (2023). Combining crocin and sorafenib improves their tumor-inhibiting effects in a rat model of diethylnitrosamine-induced cirrhotic-hepatocellular carcinoma. Cancers.

[86] Ghasemzadeh Rahbardar M, Hosseinzadeh H (2020). Therapeutic effects of rosemary (Rosmarinus officinalis ) and its active constituents on nervous system disorders. Iran J Basic Med Sci.

[87] Ghasemzadeh Rahbardar M, Amin B, Mehri S, Mirnajafi-Zadeh SJ, Hosseinzadeh H (2017). Anti-inflammatory effects of ethanolic extract of Rosmarinus officinalis L and rosmarinic acid in a rat model of neuropathic pain. Biomed Pharmacother.

[88] Rašković A, Milanović I, Pavlović N, Ćebović T, Vukmirović S, Mikov M (2014). Anti-oxidant activity of rosemary (Rosmarinus officinalis L ) essential oil and its hepatoprotective potential. BMC Complement Altern Med.

[89] Ghasemzadeh Rahbardar M, Hosseinzadeh H (2024). Therapeutic potential of hypnotic herbal medicines: A comprehensive review. Phytother Res.

[90] Nakisa N, Ghasemzadeh Rahbardar M (2022). Therapeutic potential of rosemary (Rosmarinus officinalis L ) on sports injuries: A review of patents. J Pharmacogn Res.

[91] Rahbardar MG, Amin B, Mehri S, Mirnajafi-Zadeh SJ, Hosseinzadeh H (2018). Rosmarinic acid attenuates development and existing pain in a rat model of neuropathic pain: An evidence of anti-oxidative and anti-inflammatory effects. Phytomedicine.

[92] Nakisa N, Rahbardar MG (2021). Action mechanisms of antirheumatic herbal medicines. Rheumatoid Arthritis.

[93] Alavi MS, Fanoudi S, Ghasemzadeh Rahbardar M, Mehri S, Hosseinzadeh H (2021). An updated review of protective effects of rosemary and its active constituents against natural and chemical toxicities. Phytother Res.

[94] Ghasemzadeh MR, Amin B, Mehri S, Mirnajafi-Zadeh SJ, Hosseinzadeh H (2016). Effect of alcoholic extract of aerial parts of Rosmarinus officinalis L on pain, inflammation and apoptosis induced by chronic constriction injury (CCI) model of neuropathic pain in rats. J Ethnopharmacol.

[95] Ghasemzadeh Rahbardar M, Eisvand F, Rameshrad M, Razavi BM, Tabatabaee Yazdi A, Hosseinzadeh H (2024). Carnosic acid mitigates doxorubicin-induced cardiac toxicity: Evidence from animal and cell model investigations. Iran J Basic Med Sci.

[96] Rahbardar MG, Eisvand F, Rameshrad M, Razavi BM, Hosseinzadeh H (2022). In vivo and in vitro protective effects of rosmarinic acid against doxorubicin-induced cardiotoxicity. Nutr Cancer.

[97] Rahbardar MG, Hosseinzadeh H, Martin CR, Hunter L-A, Patel VB, Preedy VR, Rajendram R (2021). Chapter 47 - Mechanisms of action of herbal antidepressants. The Neuroscience of Depression.

[98] Ghasemzadeh Rahbardar M, Hemadeh B, Razavi BM, Eisvand F, Hosseinzadeh H (2022). Effect of carnosic acid on acrylamide induced neurotoxicity: In vivo and in vitro experiments. Drug Chem Toxicol.

[99] Ghasemzadeh Rahbardar M, Hosseinzadeh H (2025). Toxicity and safety of rosemary (Rosmarinus officinalis): A comprehensive review. Naunyn Schmiedebergs Arch Pharmacol.

[100] Wei FX, Liu JX, Wang L, Li HZ, Luo JB (2008). [Expression of bcl-2 and bax genes in the liver cancer cell line HepG2 after apoptosis induced by essential oils from Rosmarinus officinalis]. Zhong Yao Cai.

[101] Melušová M, Jantová S, Horváthová E (2014). Carvacrol and rosemary oil at higher concentrations induce apoptosis in human hepatoma HepG2 cells. Interdiscip Toxicol.

[102] Tu Z, Moss-Pierce T, Ford P, Jiang TA (2013). Rosemary (Rosmarinus officinalis L ) extract regulates glucose and lipid metabolism by activating AMPK and PPAR pathways in HepG2 cells. J Agric Food Chem.

[103] Tong XP, Ma YX, Quan DN, Zhang L, Yan M, Fan XR (2017). Rosemary extracts upregulate Nrf2, Sestrin2, and MRP2 protein level in human hepatoma HepG2 cells. Evid Based Complement Alternat Med.

[104] Rodenak-Kladniew B, Castro A, Stärkel P, Galle M, Crespo R (2020). 1,8-Cineole promotes G0/G1 cell cycle arrest and oxidative stress-induced senescence in HepG2 cells and sensitizes cells to anti-senescence drugs. Life Sci.

[105] Wang T, Takikawa Y, Tabuchi T, Satoh T, Kosaka K, Suzuki K (2012). Carnosic acid (CA) prevents lipid accumulation in hepatocytes through the EGFR/MAPK pathway. J Gastroenterol.

[106] Hu Y, Zhang N, Fan Q, Lin M, Zhang C, Fan G (2015). Protective efficacy of carnosic acid against hydrogen peroxide induced oxidative injury in HepG2 cells through the SIRT1 pathway. Can J Physiol Pharmacol.

[107] Xiang Q, Ma Y, Dong J, Shen R (2015). Carnosic acid induces apoptosis associated with mitochondrial dysfunction and Akt inactivation in HepG2 cells. Int J Food Sci Nutr.

[108] Zhang X, Chen Y, Cai G, Li X, Wang D (2017). Carnosic acid induces apoptosis of hepatocellular carcinoma cells via ROS-mediated mitochondrial pathway. Chem Biol Interact.

[109] Wang X, Gupta P, Jramne Y, Danilenko M, Liu D, Studzinski GP (2020). Carnosic acid increases sorafenib-induced inhibition of ERK1/2 and STAT3 signaling which contributes to reduced cell proliferation and survival of hepatocellular carcinoma cells. Oncotarget.

[110] Wu Q, Wang X, Pham K, Luna A, Studzinski GP, Liu C (2020). Enhancement of sorafenib-mediated death of Hepatocellular carcinoma cells by Carnosic acid and Vitamin D2 analog combination. J Steroid Biochem Mol Biol.

[111] Hasei S, Yamamotoya T, Nakatsu Y, Ohata Y, Itoga S, Nonaka Y (2021). Carnosic acid and carnosol activate AMPK, suppress expressions of gluconeogenic and lipogenic genes, and inhibit proliferation of HepG2 cells. Int J Mol Sci.

[112] Wu W, Li Y, Wu X, Liang J, You W, He X (2024). Carnosic acid nanocluster-based framework combined with PD-1 inhibitors impeded tumorigenesis and enhanced immunotherapy in hepatocellular carcinoma. Funct Integr Genomics.

[113] Kong S, Xiao W, Ma T, Chen Y, Shi H, Tu J (2025). Carnosol inhibits the proliferation, migration, and invasion of hepatocellular carcinoma cells in vitro by regulating the AMPK signaling pathway. Anticancer Agents Med Chem.

[114] Ma ZJ, Yan H, Wang YJ, Yang Y, Li XB, Shi AC (2018). Proteomics analysis demonstrating rosmarinic acid suppresses cell growth by blocking the glycolytic pathway in human HepG2 cells. Biomed Pharmacother.

[115] Huang Y, Cai Y, Huang R, Zheng X (2018). Rosmarinic acid combined with adriamycin induces apoptosis by triggering mitochondria-mediated signaling pathway in HepG2 and Bel-7402 cells. Med Sci Monit.

[116] Wang L, Yang H, Wang C, Shi X, Li K (2019). Rosmarinic acid inhibits proliferation and invasion of hepatocellular carcinoma cells SMMC 7721 via PI3K/AKT/mTOR signal pathway. Biomed Pharmacother.

[117] Jerard C, Michael BP, Chenicheri S, Vijayakumar N, Ramachandran R (2020). Rosmarinic acid-rich fraction from Mentha arvensis synchronizes Bcl/Bax expression and induces Go/G1 arrest in hepatocarcinoma cells. Proc Natl Acad Sci India Sect B Biol Sci.

[118] Ozgun GS, Ozgun E (2020). The cytotoxic concentration of rosmarinic acid increases MG132-induced cytotoxicity, proteasome inhibition, autophagy, cellular stresses, and apoptosis in HepG2 cells. Hum Exp Toxicol.

[119] Jin B, Liu J, Gao D, Xu Y, He L, Zang Y (2020). Detailed studies on the anticancer action of rosmarinic acid in human Hep-G2 liver carcinoma cells: Evaluating its effects on cellular apoptosis, caspase activation and suppression of cell migration and invasion. J Buon.

[120] Chen C, Liu Y, Shen Y, Zhu L, Yao L, Wang X (2023). Rosmarinic acid, the active component of Rubi Fructus, induces apoptosis of SGC-7901 and HepG2 cells through mitochondrial pathway and exerts anti-tumor effect. Naunyn Schmiedebergs Arch Pharmacol.

[121] Elafify HS, Algendy FE, Said AM (2023). The possible impacts of rosemary and hops ethanolic extracts on hepatocellular carcinoma experimentally induced in rats. Benha Vet Med J.

[122] Abdallah HMI, El Awdan SA, Abdel-Rahman RF, Farrag ARH, Allam RM (2022). 1,8 cineole and ellagic acid inhibit hepatocarcinogenesis via upregulation of MiR-122 and suppression of TGF-β1, FSCN1, Vimentin, VEGF, and MMP-9. PLoS One.

[123] Cao W, Hu C, Wu L, Xu L, Jiang W (2016). Rosmarinic acid inhibits inflammation and angiogenesis of hepatocellular carcinoma by suppression of NF-κB signaling in H22 tumor-bearing mice. J Pharmacol Sci.

[124] Cao W, Mo K, Wei S, Lan X, Zhang W, Jiang W (2019). Effects of rosmarinic acid on immunoregulatory activity and hepatocellular carcinoma cell apoptosis in H22 tumor-bearing mice. Korean J Physiol Pharmacol.

[125] Cao W, Pan J, Mo K, Wang Z, Wei S, Yin Y (2023). Effects of gene silencing of indoleamine 2,3-dioxygenase 1 combined with rosmarinic acid on tumor immune microenvironment in H22 tumor-bearing mice. Int Immunopharmacol.

[126] Abdullaev FI, Riverón-Negrete L, Caballero-Ortega H, Manuel Hernández J, Pérez-López I, Pereda-Miranda R (2003). Use of in vitro assays to assess the potential antigenotoxic and cytotoxic effects of saffron (Crocus sativus L ). Toxicol In Vitro.

[127] Li Y, Darwish WS, Chen Z, Tan H, Wu Y, Suzuki H (2019). Identification of lead-produced lipid hydroperoxides in human HepG2 cells and protection using rosmarinic and ascorbic acids with a reference to their regulatory roles on Nrf2-Keap1 anti-oxidant pathway. Chem Biol Interact.

